# Functional Analysis of the *Brassica napus* L. Phytoene Synthase (PSY) Gene Family

**DOI:** 10.1371/journal.pone.0114878

**Published:** 2014-12-15

**Authors:** Ada López-Emparán, Daniela Quezada-Martinez, Matías Zúñiga-Bustos, Víctor Cifuentes, Federico Iñiguez-Luy, María Laura Federico

**Affiliations:** 1 Genomics and Bioinformatics Unit, Agriaquaculture Nutritional Genomic Center, CGNA, Temuco, La Araucanía, Chile; 2 Instituto de Biología Vegetal y Biotecnología, Universidad de Talca, Talca, Maule, Chile; 3 Depto. Cs. Ecológicas, Facultad de Ciencias, Universidad de Chile, Santiago, Región Metropolitana, Chile; University of Texas at Austin, United States of America

## Abstract

Phytoene synthase (PSY) has been shown to catalyze the first committed and rate-limiting step of carotenogenesis in several crop species, including *Brassica napus* L. Due to its pivotal role, PSY has been a prime target for breeding and metabolic engineering the carotenoid content of seeds, tubers, fruits and flowers. In *Arabidopsis thaliana*, PSY is encoded by a single copy gene but small PSY gene families have been described in monocot and dicotyledonous species. We have recently shown that PSY genes have been retained in a triplicated state in the A- and C-Brassica genomes, with each paralogue mapping to syntenic locations in each of the three “Arabidopsis-like” subgenomes. Most importantly, we have shown that in *B. napus* all six members are expressed, exhibiting overlapping redundancy and signs of subfunctionalization among photosynthetic and non photosynthetic tissues. The question of whether this large PSY family actually encodes six functional enzymes remained to be answered. Therefore, the objectives of this study were to: (i) isolate, characterize and compare the complete protein coding sequences (CDS) of the six *B. napus* PSY genes; (ii) model their predicted tridimensional enzyme structures; (iii) test their phytoene synthase activity in a heterologous complementation system and (iv) evaluate their individual expression patterns during seed development. This study further confirmed that the six *B. napus* PSY genes encode proteins with high sequence identity, which have evolved under functional constraint. Structural modeling demonstrated that they share similar tridimensional protein structures with a putative PSY active site. Significantly, all six *B. napus* PSY enzymes were found to be functional. Taking into account the specific patterns of expression exhibited by these PSY genes during seed development and recent knowledge of PSY suborganellar localization, the selection of transgene candidates for metabolic engineering the carotenoid content of oilseeds is discussed.

## Introduction

The first committed step of the carotenoid biosynthetic pathway, the formation of phytoene from two molecules of geranylgeranyl pyrophosphate (GGPP), is catalyzed by the enzyme phytoene synthase (PSY). Since GGPP also serves as a precursor for the synthesis of tocopherols, chlorophylls, plastoquinones and gibberellins, *PSY* gene expression is highly regulated and represents an important checkpoint for controlling the flux (of carbon) into the carotenoid biosynthetic pathway [1]–[6].

PSY is encoded by a single copy gene (*AtPSY*, At5g17230) in *Arabidopsis thaliana*, therefore, controlling the flux and responses of the pathway is limited to regulating this unique enzyme [5]. Most plant species, however, contain small *PSY* gene families composed of two or three members [7]–[12]. In these plant species, subfunctionalization of *PSY* gene expression has been described mainly between photosynthetic and non photosynthetic tissues [7], [8], [13], [14]. This subfuntionalization has allowed for *PSY* overexpression in flowers, fruits, seeds or tubers without the detrimental effects that excessive carotenoid accumulation throughout the plant would have caused on photosynthesis [9] since carotenoids and chlorophylls are required to accumulate in a defined stoichiometric ratio in chloroplasts. In addition, it has been shown that specific *PSY* paralogues can mediate the stress-induced production of abscisic acid (ABA) in roots [11], [12], [15].

*Brassica napus* L. (AACC; *n* = 19) is an allotetraploid species originated from the relatively recent hybridization of two base diploid genomes [16]. These diploid parental species, *Brassica rapa* L. (AA; *n* = 10) and *Brassica oleracea* L. (CC; *n* = 9), are considered ancient polyploids, each one of them composed of three paralogous “Arabidopsis-like” subgenomes [17]–[20]. These paralogous subgenomes are by no means equal, exhibiting variable gene content with interspersed gene losses and insertions [21]–[25]. We have recently reported that PSY genes have been retained in a triplicated state in both A- and C- Brassica diploid genomes and that *B. napus* bears the largest plant PSY gene family described to date [14]. All Brassica PSY gene family members are expressed, exhibiting overlapping redundancy and signs of subfunctionalization. In *B. napus*, expression of homeologues *BnaC.PSY.a* and *BnaA.PSY.b* were detected in all tissues, but homeologous gene pairs *BnaA.PSY.d*/*BnaC.PSY.f* and *BnaA.PSY.c*/*BnaC.PSY.e* exhibited preferential expression in chloroplast- and chromoplast-rich tissues, respectively [14].

PSY genes have been retained in Brassica species in spite of the well characterized fractionation of the subgenomes [25]; a process by which a genome tends to return to its original gene complement after a genome duplication event [26]. Remarkably, only 10% of *A. thaliana* predicted gene models have been found to be retained as syntenic orthologues in the three subgenomes of the sequenced *B. rapa* and *B. oleracea* genomes [19], [20]. Undoubtedly, the retention of six PSY genes in *B. napus* underpins the importance of this enzyme and could be at least partially explained by the selective advantage provided by increased levels of gene product in floral organs [14].

PSY activity has been shown to be a rate-limiting factor for carotenoid biosynthesis in non-photosynthetic tissues [27]. In *B. napus*, this limitation was demonstrated through pioneer work on metabolic engineering when the sole over-expression of a bacterial phytoene synthase gene (*CrtB*) in seeds raised β-carotene levels 50-fold [28]. The use of different *PSY* transgene sources, however, can enhance carotenoid content to various degrees, as observed in transgenic rice callus and grains [29], highlighting the importance of *PSY* transgene efficacy. In this context, it was important to investigate whether all six *B. napus* PSY genes were functional and determine which ones could represent better transgene alternatives to metabolically engineer the carotenoid content of oilseed crops. Therefore, the objectives of the present work were to: isolate, characterize and compare the complete protein coding sequences (CDS) of the *B. napus PSY* genes; to model their predicted tridimensional enzyme structures; to determine whether they all encode functional phytoene synthases using a heterologous complementation system and to evaluate their specific expression patterns during seed development.

## Materials and Methods

### Plant materials and nucleic acid isolation

*Brassica napus* cv. Westar plants were grown in a controlled greenhouse under 16-h-day/8-h-night cycle. For genomic DNA (gDNA) extraction, flower buds were harvested and lyophilized. DNA isolation was conducted following the CTAB procedure described by Kidwell and Osborn [30]. For total RNA extraction, cotyledons; seedlings with 3–4 true leaves; young leaves from mature plants; seeds and petals were harvested, immediately frozen in liquid nitrogen and stored at −80°C.

### Rapid amplification of cDNA ends (RACE) of *B. napus* PSY genes

Total RNA was extracted using RNA-Solv Reagent (Omega Bio-Tek, GA, USA) following the manufacturer's instructions. Total RNA (2 µg) from all tissues were treated with RQ1 DNAse (Promega, WI, USA) and first strand cDNA synthesis was carried out using an oligo(dT) primer and M-MuLV Reverse Transcriptase (New England BioLabs, MA, USA) as previously described [14]. Several *BnaX.PSY*-specific primers (S1 Table in S1 File) were designed in order to clone the 5′and 3′ cDNA ends of six *B. napus* PSY genes using the GeneRacer kit (Invitrogen, CA, USA). Total RNAs from leaf, petal and seedling tissues were used to synthesize RACE-ready cDNA as described in Federico et al [31] with minor modifications. The resulting PCR fragments were gel-purified (E.Z.N.A. Gel Extraction Kit, Omega Bio-Tek, GA, USA) and cloned into the StrataClone Blunt vector (Agilent Technologies, CA, USA). After *E. coli* transformation, several colonies per clone were sequenced.

### Cloning of *B. napus PSY* protein coding sequences

Based on a previous report [14], oligonucleotide primers (Bna.PSY.15F and Bna.PSY.8R, S1 Table in S1 File) were used to amplify *BnaC.PSY.a* (GenBank JF920037) and *BnaA.PSY.b* (GenBank JF920038) complete coding sequences (CDS) with KOD Hot Start DNA Polymerase (Novagen, WI, USA) from leaf, petal and seed *B. napus* cDNAs. Specific PCR products (1290 bp) were cloned into the pGEM-T vector (Promega, WI, USA) and several colonies per clone were sequenced to confirm identity. Specific oligonucleotide primers (S1 Table in S1 File) were designed and used to amplify the remaining four CDS (*BnaX.PSY.c-f*) using Go Taq DNA polymerase (Promega) from petal and cotyledon *B. napus* cDNAs. Specific PCR fragments were cloned in pCR2.1-TOPO vector with the TOPO TA cloning kit (Invitrogen) and several colonies per clone were sequenced to confirm identity.

### *B. napus PSY* protein coding sequence analysis

Sequence reads from each *BnaX.PSY* clone including cDNA, RACE and genomic DNA amplifications were assembled using the contig tool of DNA Baser Sequence Assembler v3.x (Heracle BioSoft SRL Romania, http://www.DnaBaser.com) and compared to *BnaX.PSY.a-f* GenBank accessions (JF920037-JF920042). Nucleotide and protein sequence alignments of complete CDS were performed using ClustalW2 [32]. The Arabidopsis *PSY* gene (At5g17230) was included as a reference. The presence of chloroplast transit peptides was predicted using the ChloroP 1.1 server [33]. Protein conserved domains were identified using the NCBI CDD tool [34]. Nucleotide replacement (Ka) and synonymous (Ks) substitutions were estimated using DNASP5 [35]. The evolutionary history was inferred using the Neighbor-Joining method [36] with a 500-bootstrap replication support [37] using MEGA4 software [38]. Tree branches corresponding to partitions reproduced in less than 50% bootstrap replicates were collapsed. The evolutionary distances were computed using the Poisson correction method [39]. All positions containing gaps and missing data were eliminated from the dataset (complete deletion option). There were a total of 321 positions in the final dataset. Genbank accessions of PSY enzymes used in the analysis are as follows: Arabidopsis (AtPSY, AAA32836), cassava (MePSY1, ACY42666; MePSY2; ACY42670), maize (ZmPSY1, P49085; ZmPSY2, AAQ91837; ZmPSY3, ABC75827), pepper (CaPSY1, ACE78189.1), rice (OsPSY1, AAS18307; OsPSY2, AAK07735; OsPSY3, ABC75828), sorghum (SbPSY1, AAW28996; SbPSY2, XP002442578; SbPSY3, AAW28997) and tomato (SlPSY1, P08196.2; SlPSY2, ABV68559.1; SlPSY3, XP_004228928.1). The cassava MePSY3 protein sequence was obtained from Phytozome (http://www.phytozome.net/cassava.php) as described in Arango *et al* [12].

### Molecular Modeling of *B. napus* PSY proteins

Protein structure predictions were made by a combination of *ab initio* folding and threading the amino acid sequence of each *BnaX.PSY* gene without its chloroplast transit peptide onto multiple squalene synthase and carotenoid dehydrosqualene synthase templates using the I-TASSER online server [40]. The threaded structures were evaluated on the ProSA server [41], [42]. To reduce the steric strains present in the structures, energy minimization and molecular dynamics simulations of 2 ns were performed for each modeled structure using the NAMD software [43]. During molecular dynamics simulations, each system was solvated into a TIP3P water box and neutralized with NaCl to mimic biological conditions.

### PSY functional complementation test in *E. coli*

To test whether each of the six *B. napus* PSY proteins have phytoene synthase activity, a heterologous complementation assay was carried out. Briefly, two *Escherichia coli* BL21-Gold strains were used in the complementation assay (S1A Figure in S1 File). One β-carotene producer strain (DS1B) transformed with plasmid pDS1B, a pBAD33 vector carrying *Erwinia uredovora* carotenogenic genes *crtE*, *crtB*, *crtI*, *crtY* and *CrtX* and a non-producer strain (DS1B-Δ*crtB*) transformed with plasmid pDS1B-Δ*crtB* which has a deletion of the *Eu crtB* gene [44]. Strain DS1B accumulates β-carotene and served as a positive control. Strain pDS1B-Δ*crtB* transformed with an empty pETblue1 expression vector (Novagen, USA) served as negative control. Each *B. napus* PSY coding sequence without its corresponding signal peptide (S2 Figure in S1 File) and encoding an ATG start codon was amplified using KOD Hot Start DNA Polymerase (Novagen, USA) from corresponding minipreps using specific primers (S1 Table in S1 File). PCR products were gel-purified (E.Z.N.A. Gel Extraction Kit, Omega Bio-Tek, GA, USA) and cloned into the *EcoRV* site of pETblue-1 (S1B Figure in S1 File). Each resulting *B. napus* PSY pETblue-1 expression vector was transformed into strain pDS1B-Δ*crtB* using an ECM600 electroporation system (BTX, MA, USA) to test for enzyme activity. Transformants were grown in 15 ml of Luria-Bertani (LB) medium containing appropriate antibiotics (100 µg ml^−1^ ampicillin, 34 µg ml^−1^ chloramphenicol) overnight at 37°C. Cultures (1 ml) were used to inoculate 100 ml of supplemented LB and grown at 37°C until they reached an optical density of 0.5–0.8 at 600 nm. Then, they were grown at 30°C for 64 h in the dark to maximize carotenoid production. At that time, 15 ml were used to calculate the dry weight of the cultures. A total of 80 ml were used to extract carotenoids. Cultures were prepared in triplicates. Extraction proceeded with centrifugation at 3000 g for 20 min, pellets were washed with water and carotenoids extracted three times with acetone and once with petroleum ether. Extracts were concentrated to dryness under a stream of nitrogen, resuspended in acetone, filtered and subjected to HPLC analysis as described previously [45]. Beta-carotene was identified by comparing retention times and absorption pattern spectra with that of a natural standard (DHI, Denmark). Differences in β-carotene content were established using Tukey's Honestly Significant Difference (HSD) test at (p<0.05) after analysis of variance using JMP Genomics 6.1.

### RT-PCR analysis during *B. napus* seed development

For reverse transcriptase polymerase chain reaction (RT-PCR) analysis, total RNA from developing seeds collected at 20, 30, 45 and 60 days post anthesis (dpa) and leaves were extracted using the Absolutely RNA RT-PCR Miniprep Kit (Agilent Technologies, CA, USA) according to the manufacturer's instructions. cDNA synthesis was performed using the SuperScript VILO cDNA Synthesis Kit (Invitrogen, Ca, USA) from 2 µg of total RNA in a 30 µl reaction. cDNA synthesis was tested by PCR amplification of the *B. napus* 18S gene (EX119428) using the Bna.18S primer pair (S1 Table in S1 File). Absence of gDNA contamination was tested by PCR amplification of the *B. napus* Actin gene (AF111812) with the BnActin primer pair (S1 Table in S1 File). This primer pair generates a predicted 725-bp fragment for cDNA and a 900-bp fragment for gDNA due to presence of an intron. RT-PCRs were performed using GoTaq Flexi DNA Polymerase (Promega, WI, USA). Amplification started with a 95°C denaturation step (5 min), followed by 40 cycles of 30 sec at 95°C, 30 sec at 55–57°C and 1 min at 72°C, with a final 72°C extension of 5 min. RT-PCR products were run and visualized on ethidium bromide-stained 1% agarose gels. Primer specificity was checked by testing primer pairs using plasmids containing each of the six *B. napus* PSY genes as templates and running the resulting PCR products along with RT-PCR products on single stranded conformation polymorphism (SSCP) gels (S3 Figure in S1 File). For SSCP gels, 20 µL of PCR products were gel-purified (E.Z.N.A. Gel Extraction Kit, Omega Bio-Tek, GA, USA) and eluted in 20 µL. Purified products (6 µL) were then mixed with 12 µL of SSCP loading buffer (95% formamide, 10 mM NaOH, 0,25% (w/v) xylene cyanol, 0,25% (w/v) bromophenol blue). For plasmid controls, 3 µL were mixed with 15 µL of loading buffer. SSCP analysis was essentially as described in [14].

### Accessions

*B. napus PSY* protein coding sequences described in this paper have been submitted to GenBank under the following accession numbers: *BnaC.PSY.a*, KF297333; *BnaA.PSY.*b, KF297330; *BnaA.PSY.c*, KF297331; *BnaA.PSY.d*, KF297332; *BnaC.PSY.e*, KF297334 and *BnaC.PSY.f*, KF297329.

## Results

### Sequence analysis of *B. napus* PSY genes

**Figure 1:**
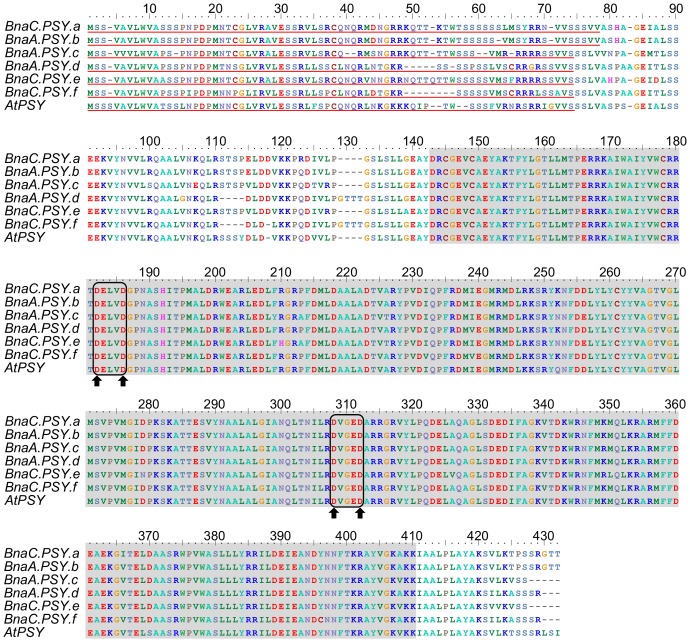
Multiple alignment of *B. napus* PSY enzymes. All PSY enzymes contain a predicted chloroplast transient peptide at the N-terminal region (underlined in red) and a conserved trans-isoprenyl diphosphate synthase domain (highlighted in grey). The putative active site (DXXXD) is boxed in black. Black arrows indicate the presence of four conserved aspartate residues.

Gene cloning, DNA-SSCP and Southern blot analyses revealed the existence of at least six PSY homologues in *B*. *napus* [14]. These PSY genes are expressed, exhibiting overlapping redundancy and signs of subfunctionalization among photosynthetic and non photosynthetic tissues [14]. However, the question of whether this large PSY family actually encoded six functional enzymes remained to be answered. Therefore, we extended our previous work by cloning, characterizing and comparing the complete protein coding sequences (CDS) of these six *B. napus* PSY genes. Using this complete CDS information, we were able to re-analyze their nucleotide and amino acidic sequences (S2 Figure in S1 File; Fig. 1). As illustrated in Fig. 1, *B. napus* PSY genes encode proteins of similar length (414–424 aa) and contain characteristic motifs: a plastid transit peptide (TP), a conserved trans-isoprenyl diphosphate domain (trans-IPP) and a putative phytoene synthase active site (DXXXD) with 4 conserved aspartate residues [46]. This conserved trans-IPP domain is present in phytoene synthases, which catalyze the head to head (1′-1) condensation of two molecules of GGPP to produce phytoene. Protein sequence identity among the six PSY homologues ranged from 87.3 to 98.8 percent (S2 Table in S1 File) with most of the variability being found at the N-terminal regions, which encode TPs. When PSY proteins were evaluated without their TPs, sequence identity percentages among homologues increased, ranging from 91.6 to 99.4% (S2 Table in S1 File).

**Figure 2:**
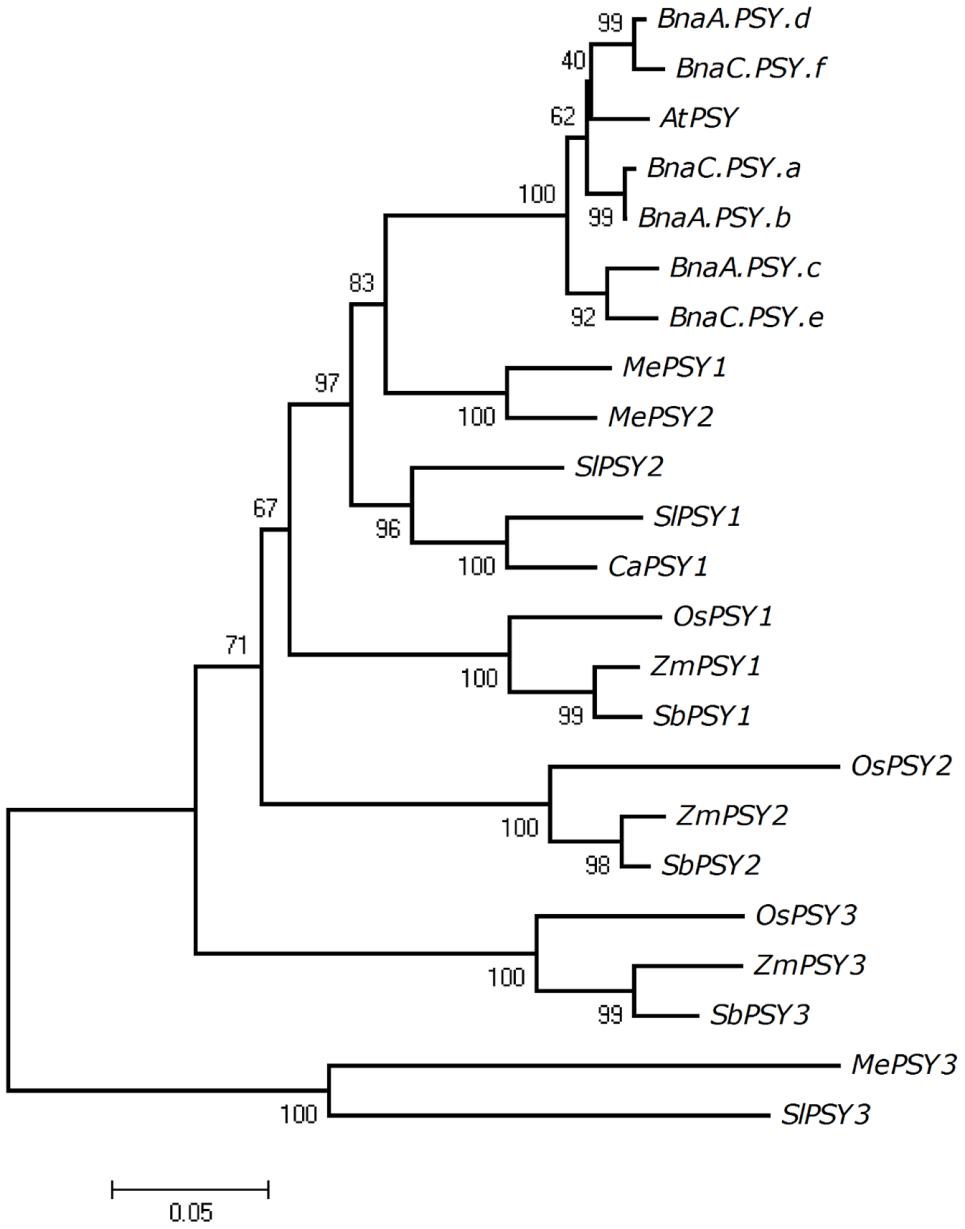
Phylogenetic relationship of *B. napus* and selected monocot and dicot PSY enzymes. The evolutionary history was inferred using the neighbor-joining method using MEGA4 [38]. The percentage of replicate trees in which the associated taxa clustered together in the 500-bootstrap replication test is shown next to the branches. The tree is drawn to scale, with branch lengths in the same units (number of amino acid substitutions per site) as those of the evolutionary distances used to infer the phylogenetic tree. The evolutionary distances were computed using the Poisson correction method [39]. Arabidopsis (AtPSY, AAA32836), cassava (MePSY1, ACY42666; MePSY2; ACY42670), maize (ZmPSY1, P49085; ZmPSY2, AAQ91837; ZmPSY3, ABC75827), pepper (CaPSY1, ACE78189.1), rice (OsPSY1, AAS18307; OsPSY2, AAK07735; OsPSY3, ABC75828), sorghum (SbPSY1, AAW28996; SbPSY2, XP002442578; SbPSY3, AAW28997) and tomato (SlPSY1, P08196.2; SlPSY2, ABV68559.1; SlPSY3, XP_004228928.1). The cassava MePSY3 protein sequence was obtained from Phytozome (http://www.phytozome.net/cassava.php) as described in Arango *et al* [12].

In order to investigate the evolutionary relationship between these *B. napus* PSY proteins and other plant PSYs whose functionality has been previously established [7], [8], [10]–[13], [15], [47], a phylogenetic analysis was conducted using 17 monocot and dicot PSYs. The phylogenetic tree showed that all *B. napus* PSY proteins cluster together with the Arabidopsis PSY (At5g17230) and that they are more closely related to dicot PSYs (MePSY1, MePSY2, SlPSY1, SlPSY2 and CaPSY1) than the extensively characterized monocot PSYs (Fig. 2). As expected, monocot PSYs formed distinct clades that exhibit different roles *in planta*, with stress-related PSYs (OsPSY3, ZmPSY3, SbPSY3) [11], [15] clustering together (Fig. 2).

### Tridimensional enzyme structure prediction of *B. napus* PSY genes

**Figure 3:**
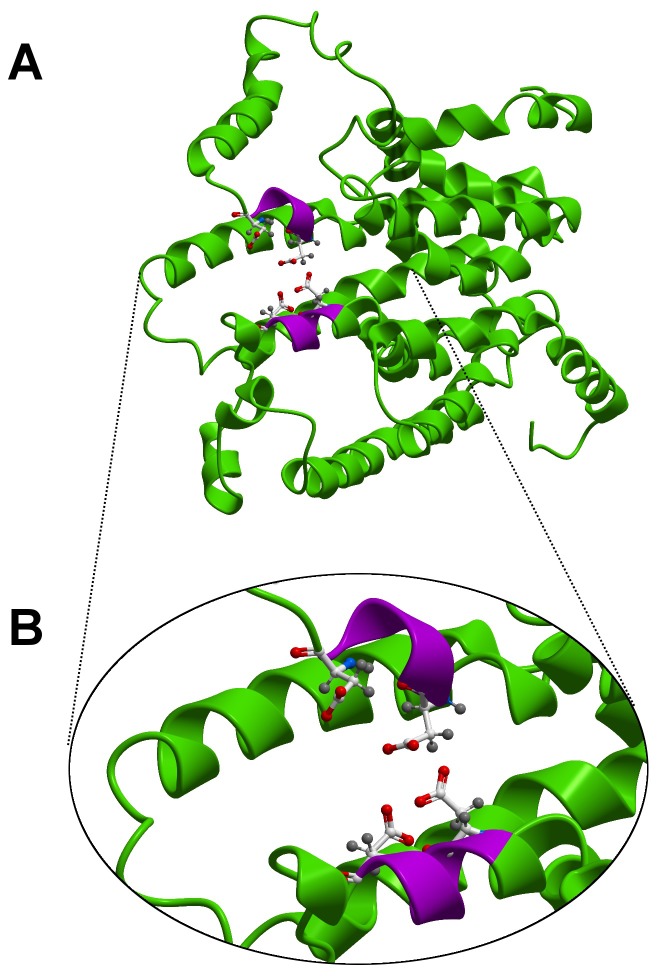
*B. napus* PSY tridimensional enzyme structure prediction. A. Modeled structure of the BnaC.PSY.a protein, an alpha-helix rich structure. B. Putative active site. Alpha-helices are shown in green, the putative active site (DXXXD) in purple with the four conserved aspartate residues shown as licorice representations [49].

Tridimensional (3D) structures of PSYs have not been determined experimentally and thus, modeling was performed using 3D information from enzymes that share conserved protein domains and catalyze similar enzymatic reactions (S4 Figure in S1 File). Like PSYs, squalene synthases (SQS) and carotenoid dehydrosqualene synthases also catalyze the head to head condensation of prenyl diphosphates to form linear terpenes [46], [48]. Our protein modeling predictions showed that the six *B. napus* PSY proteins are alpha-helix rich structures, with anti-parallel alpha-helixes forming a large central cavity (S5 Figure in S1 File). As an example, the tridimensional structure prediction for the BnaC.PSY.a enzyme is shown in Fig. 3. The two conserved aspartate-rich domains, DELVD and DVGED, localize to two alpha helixes on opposite walls of the central cavity where the condensation of two molecules of GGPP is predicted take place to produce phytoene. Amino acid differences between the six *B. napus* PSY proteins do not alter or affect the structure of this putative active site region (S3 Figure in S1 File). Subtle differences in amino acid content found in this region, however, could have profound effects on enzyme activity as seen in other plant PSYs [50], [51].

### Functional characterization of *B. napus* PSY genes

**Figure 4:**
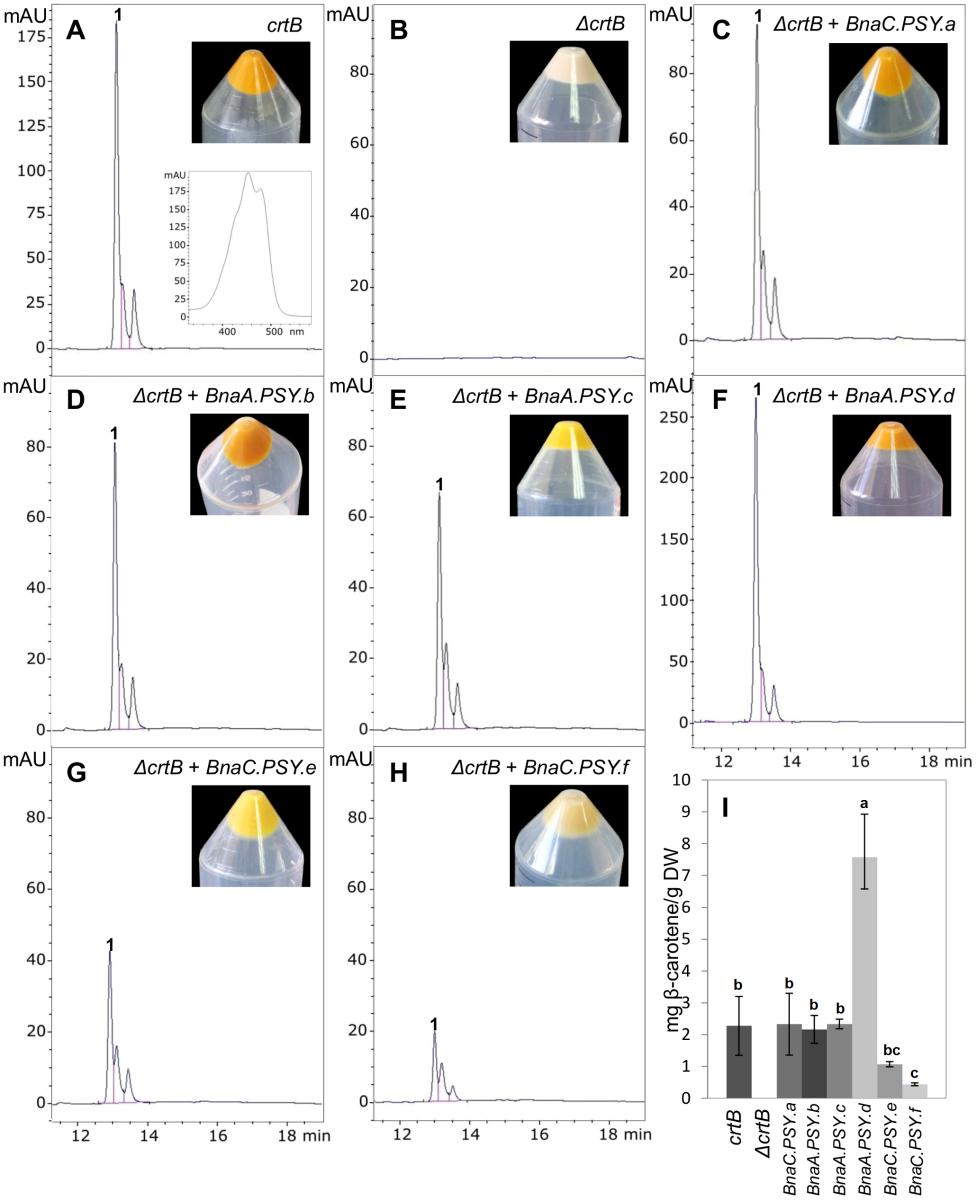
Functional complementation of *B. napus* PSY genes. *E. coli* cells were transformed with: (A) pDS1B, a pBAD33 vector carrying *E. uredovora* carotenogenic genes *crtE*, *crtB*, *crtI*, *crtY* and *CrtX*; (B) pDS1B-Δ*crtB* which has a deletion of the *Eu crtB* gene + pETBlue1 (empty vector); (C) pDS1B-Δ*crtB* + pETBnaC.PSY.a; (D) pDS1B- Δ*crtB* + pETBnaA.PSY.b; (E) pDS1B- Δ*crtB* + pETBnaA.PSY.c; (F) pDS1B- Δ*crtB* + pETBnaA.PSY.d; (G) pDS1B- Δ*crtB* + pETBnaC.PSY.e; and (H) pDS1B- Δ*crtB* + pETBnaC.PSY.f and HPLC chromatograms obtained at 450 nm are shown for each transformation. The spectral fine spectrum for beta carotene (peak 1) is shown as an example in the control panel (A). The amount (mg) of β-carotene produced by each complementation assay expressed as per gram of dry weight (DW) is shown in (I). Bars represent standard deviation calculated from three replications. For each vector combination, different letters indicate significant differences at p<0.05 determined by Tukey's HSD test.

The existence of engineered strains of *E. coli* carrying different combinations of genes of the carotenoid biosynthetic pathway provide an extremely useful and practical system to assay for enzyme function [44], [52], [53]. Six *B. napus* PSY CDS, lacking their corresponding plastid TPs, were subcloned into the pETBlue1 expression vector (Novagen, USA) and transformed into *E. coli* harboring pDS1B-Δ*crtB* (S1 Figure in S1 File) to test for phytoene synthase activity [44]. *E. coli* cells produced the final end product of the transformed pathway, β-carotene, in the presence of the bacterial *PSY* gene *CrtB* (Fig. 4A) but not when the empty pETBlue1 vector was cotransformed with pDS1B-Δ*crtB* (Fig. 4B). These *E. coli* cells, DS1B and DS1B-Δ*crtB*, served as positive and negative controls, respectively. Significantly, *E. coli* cells harboring pDS1B-Δ*crtB* and cotransformed with each of the six *B. napus* PSY genes were capable of producing β-carotene (Fig. 4C-H). In all cases, HPLC analysis of carotenoid extracts from the *E. coli* cell cultures showed a β-carotene peak, which matched in retention time and spectrum with that observed for the positive control (Fig. 4A). Thus, we were able to confirm that this large PSY gene family encodes functional enzymes with *BnaA.PSY.d* producing the largest accumulation of β-carotene under this heterologous complementation system conditions (Fig. 4I).

### *B. napus* PSY gene expression analysis during seed development

**Figure 5:**
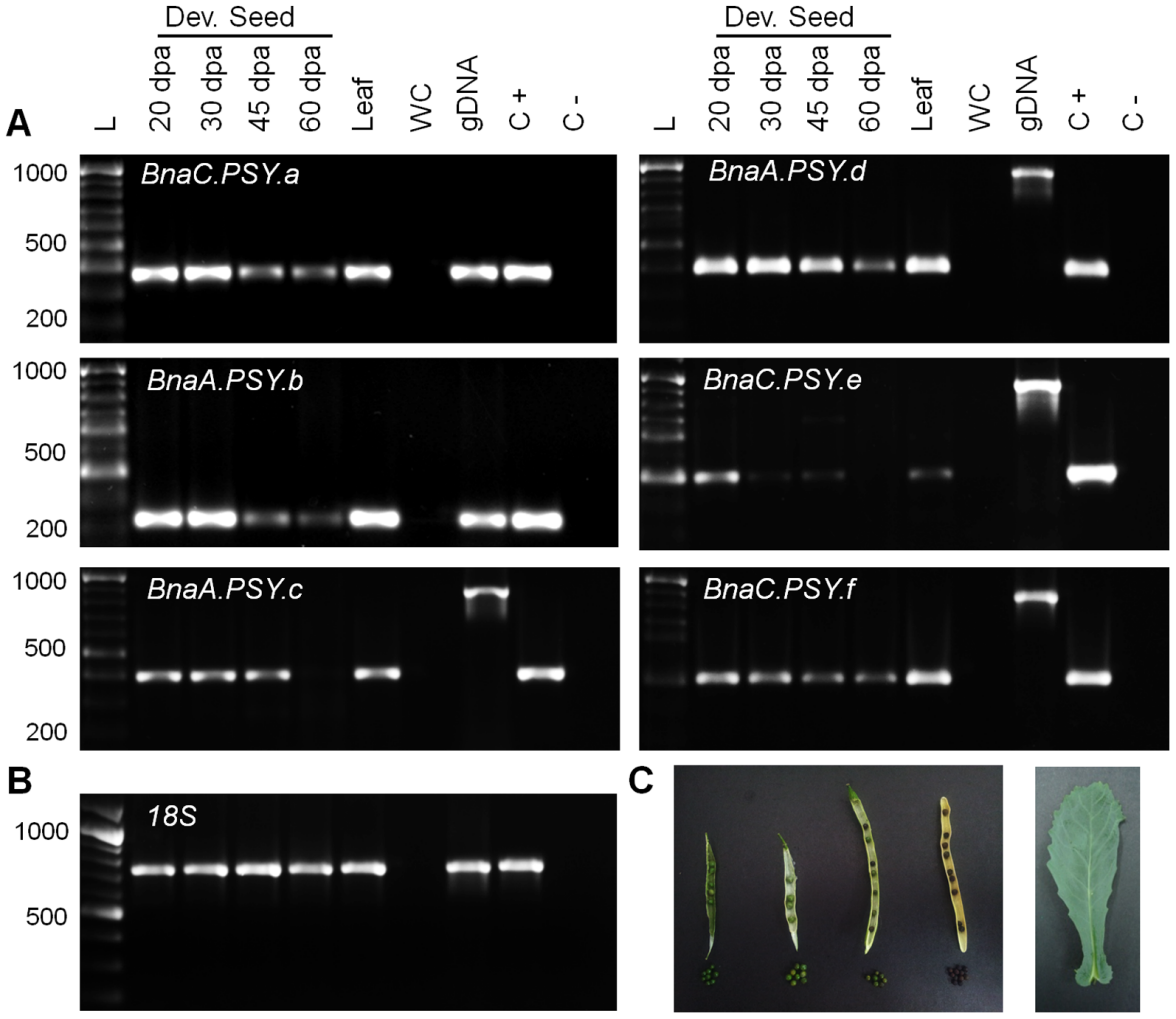
*B. napus* PSY gene expression during seed development. A. *BnaX.PSY* gene expression was determined by RT-PCR using homologue-specific primers. B. *B*. *napus 18S* gene expression (loading control). C. Sampling stages of *B. napus* seed development (from left to right: 20, 35, 40 and 60 days post anthesis) and leaf tissue. L: 100 bp ladder; WC: water control; gDNA: *B*. *napus* gDNA control; C+: PSY homologue-specific plasmid controls (positive); C-: PSY homoelogue plasmid controls (negative). RT-PCR (40 cycles) was performed in two biological replicates, only one is shown for simplicity.

Metabolic engineering of high carotenoid seed oil content could benefit from the overexpression of a PSY gene which naturally exhibits seed-specific expression, as seen with maize PSY1 [10], [13], [29]. Therefore, the expression of each *B. napus* PSY was assessed by RT-PCR using homologue-specific primers at four different stages of seed development. Transcripts of all six members of the PSY family were detected in developing seeds, primarily at early stages (Fig. 5). This observed expression pattern agreed with previous studies that indicated a critical stage of *B. napus* seed development in regards to cell proliferation, oil deposition and carotenoid accumulation starting at around 20 dpa [54]-[56]. Homologue pairs *BnaC.PSY.a/BnaA.PSY.b* and *BnaA.PSY.d/BnaC.PSY.f* could be detected at 20, 30, 45 and 60 dpa under tested conditions (Fig. 5). Interestingly, *BnaC.PSY.e*, one of the two homoelogues previously shown to be preferentially expressed in petals [14], was the PSY gene least expressed during seed development (Fig. 5).

## Discussion

Carotenoids are isoprenoid compounds synthesized in plant cell plastids. They fulfill a variety of plant functions during photosynthesis, pollination, seed dispersal and stress [57]-[60]. Certain carotenoids also fulfill nutritional requirements in humans since they act as precursors of vitamin A (β-carotene) and help prevent age-related macular degeneration (lutein, zeaxanthin) and other diseases [61]–[63]. Classical breeding and metabolic engineering efforts have been mainly focused on increasing β-carotene content in edible plant tissues to alleviate vitamin A deficiency [64]–[68]. Successful stories of such efforts include the development of Golden Rice [29], [67]. However, predictable control of carotenoid biosynthesis has not been accomplished to date. Specific details about pathway bottlenecks, enzyme suborganellar localizations, metabolon assembly and activity are only partially known and could also be tissue and species specific [69].

Overexpression of bacterial and plant PSYs revealed that PSY activity could be rate-limiting for increasing carotenoid content in non-photosynthetic tissues [27]–[29], with certain PSY transgenes being more effective than others in promoting carotenoid accumulation in transgenic plants [29], [67]. Although the reason behind this different efficiency is not known [29], [69], it has been suggested that overexpressing a plant PSY gene in transgenic *B. napus* could result in higher levels of seed oil carotenoids than those obtained overexpressing *CrtB* [29]. Therefore, the importance of this study is two-fold. First, we have expanded our previous work demonstrating that all six members of the *B. napus* PSY gene family encode active phytoene synthases. Second, we provided sequence and functional information to help select transgene candidates for metabolic engineering the carotenoid content of oilseeds, including *B. napus*.

Carotenoid biosynthesis starts with the production of phytoene, a colorless carotenoid. This reaction is catalyzed by PSY, which is encoded by at least six different nuclear genes in *B. napus* (S2 Figure in S1 File). This gene family encodes enzymes with characteristic PSY motifs (Fig. 1) and a tridimensional structure which bears a putative PSY active site (Fig. 3, S5 Figure in S1 File). The highest level of sequence divergence among *B. napus* PSY proteins was found at the N-terminal TP region (Fig. 1) as previously observed in these Brassica and other PSY proteins [9], [10], [12], [14], [15]. In spite of this high sequence variability, TPs are recognized and bound by protein import complexes (translocons) in the outer (Toc) and inner (Tic) envelope membranes of plastids [70] successfully targeting nuclear-encoded proteins to plastids [71]. Different translocons (encoded by homologous genes) are assembled in plastids of different tissue types (e.g. photosynthetic vs. non-photosynthetic) and developmental stages [72]. No functional prediction, however, is currently possible solely based on TP sequence information.

The level of replacement and synonymous site nucleotide divergence ratio (Ka/Ks) suggest that all *B. napus* PSYs are likely undergoing purifying selection with Ka/Ks values lower than 0.28 for all gene pairs tested, which strongly indicates that these PSY proteins have evolved under functional constraint (S3 Table in S1 File). Even though *in silico* predictions (Fig. 3, S5 Figure in S1 File) indicated that these *B. napus* PSYs are likely active enzymes, only their functional characterization could confirm the ability of each of these PSYs to act as phytoene synthase. In fact, the use of a heterologous complementation system confirmed that all PSY genes could complement phytoene synthase deficient *E. coli* strains and produce β-carotene (Fig. 4). The amount of β-carotene produced by individual *B. napus* PSY genes varied, with *BnaA.PSY.d* producing the largest accumulation of β-carotene (Fig. 4I). Albeit this differential accumulation of β-carotene in *E. coli* clones transformed with individual *B. napus* PSY genes could be the result of a variety of factors (e.g. protein stability, folding and/or solubility) related to the heterologous system itself, discrete differences in amino acid sequence found between the different PSY homologues could also be the cause. In cassava PSY2, for example, a single nucleotide polymorphism (SNP, D_191_) leading to a single amino acid substitution (alanine to aspartic acid at position 191) was associated to the higher carotenoid content of yellow cassava roots [50]. Similarly, a mutation causing a proline to leucine substitution (P192L) in tomato PSY1 is linked to delayed carotenoid accumulation in the fruit, possibly due to reduced PSY enzymatic activity [73].

Arabidopsis and *B. napus* PSYs together with evaluated dicotyledonous PSY1 and PSY2 enzymes were found to be more related to cereal PSY1s which are preferentially expressed in seeds [10], [13]. Cassava and tomato PSY3s (MePSY3, SlPSY3), which were both discovered by homology searches due to their virtually undetectable gene expression levels, formed a separate branch [12], [74]. Interestingly, all six *B. napus* PSY genes were found to be expressed in seeds with homoelogue pairs *BnaC.PSY.a/BnaA.PSY.b* and *BnaA.PSY.d/BnaC.PSY.f* being detected at all developmental stages (Fig. 5). Taken together, the functional characterization of this *B. napus* PSY family (Fig. 4I, Fig. 5) indicates that *BnaA.PSY.d* could be the first transgene candidate of choice to enhance carotenoid content in oilseeds, followed by *BnaC.PSY.a*, *BnaA.PSY.b*, *BnaA.PSY.c* and *BnaC.PSY.f*.

Recently, the study of PSY suborganellar localization in maize cells revealed that different PSY1 allelic variants localize to distinct plastid compartments, underpinning the importance of enzyme and metabolome localization [51], [69]. Together with maize PSY2 and PSY3, rice and Arabidopsis PSYs, these six *B. napus* PSY enzymes posses serine (S) and proline (P) residues at positions equivalent to asparagine (N_168_) and threonine (T_257_) in maize PSY1, suggesting that they most likely localize to plastoglobuli. In addition, they also carry alanine (A) at an equivalent position to that of cassava PSY2 white allele, which could be exchanged for the aspartic acid (D) present in the yellow allele [50]. This A-to-D approach resulted in a 3-fold increase of AtPSY specific activity *in vitro* [50]. In this complex scenario, it will be worth engineering a series of BnaA.PSY.d mutagenized enzymes for seed-specific overexpression in transgenic *B. napus* and other related oilseed crops like *Camelina sativa*. Undoubtedly, a more detailed characterization of PSY and other carotenoid biosynthetic gene families will help to better design metabolic engineering strategies.

## Supporting Information

S1 File Contains the following files: **S1 Figure.** PSY Heterologous Complementation System. A. Two *E. coli* BL21-Gold strains were used, a β-carotene producer strain (DS1B) transformed with plasmid pDS1B, a pBAD33 vector carrying *Erwinia uredovora* carotenogenic genes *crtE*, *crtB*, *crtI*, *crtY* and *CrtX* and a non-producer strain (DS1B-Δ*crtB*) transformed with plasmid pDS1B-Δ*crtB* which has a deletion of the *Eu crtB* gene. B. Six pETBlue1-BnaX.PSY vectors were used, each carrying a *B. napus* PSY homologue, without its corresponding signal peptide, cloned into the EcoRV site. **S2 Figure**. Multiple nucleotide *BnaX.PSY* sequence alignment. **S3 Figure**. Homologue-specific PCR primer control reactions. A. Primer specificity was tested by PCR using plasmids containing each of the six *B. napus* PSY genes. Primers BnaC.PSY.a and BnaA.PSY.b could not be tested against BnaA.PSY.d and BnaC.PSY.f (clones did not include 5′UTRs). **B**. SSCP analysis of *BnaC.PSY.a* RT-PCR reactions show that only two strands exhibiting the same exact pattern as the *BnaC.PSY.a* plasmid control are present, confirming primer specificity. **C**. SSCP analysis of *BnaA.PSY.b* RT-PCR reactions show that only two strands exhibiting the same exact pattern as the *BnaA.PSY.b* plasmid control are present, confirming primer specificity. L: 100 bp ladder; BnaX.PSY.a-f: plasmid DNA controls; gDNA: *B*. *napus* genomic DNA control; WC: water control; ND. Not determined; 20 dpa: seed cDNA 20 days post anthesis; Leaf: leaf cDNA. **S4 Figure**. Alignment of *B. napus* PSY proteins with squalene synthase and carotenoid dehydrosqualene synthase templates. 2zcs: *Staphylococcus aureus* Dehydrosqualene synthase complexed with BPH-700; 3acx: *Staphylococcus aureus* Dehydrosqualene synthase complexed with BPH-673; 4e9u: *Staphylococcus aureus* Dehydrosqualene synthase complexed with thiocyanate inhibitor; 2zco: *Staphylococcus aureus* Dehydrosqualene synthase; 4hdl: PpcA F15L mutant from *Geobacter sulfurreducens*; 3vj8: *Homo sapiens* Squalene synthase. **S5 Figure**. Tridimensional enzyme structure prediction of *B. napus* PSY homoelogous pairs. Alpha-helices are shown in green and the putative active site (DXXXD) in purple with the four conserved aspartate residues shown as licorice representations [49]. **S1 Table**. Oligonucleotide primers used in this study. **S2 Table**. BnaX.PSY protein sequence identity (%). **S3 Table**. Ka/Ks.
(DOCX)
