# Supplementary material for: Functional Analysis of the Brassica napus L. Phytoene Synthase (PSY) Gene Family
Source: PLoS One. 2014 Dec 15;9(12):e114878. doi: 10.1371/journal.pone.0114878 (PMC4266642; doi:10.1371/journal.pone.0114878)
Supplement: S1 File — Contains the following files: S1 Figure. PSY Heterologous Complementation System. A. Two E. coli BL21-Gold strains were used, a β-carotene producer strain (DS1B) transformed with plasmid pDS1B, a pBAD33 vector carrying Erwinia uredovora carotenogenic genes crtE, crtB, crtI, crtY and CrtX and a non-producer strain (DS1B-ΔcrtB) transformed with plasmid pDS1B-ΔcrtB which has a deletion of the Eu crtB gene. B. Six pETBlue1-BnaX.PSY vectors were used, each carrying a B. napus PSY homologue, without its corresponding signal peptide, cloned into the EcoRV site. S2 Figure. Multiple nucleotide BnaX.PSY sequence alignment. S3 Figure. Homologue-specific PCR primer control reactions. A. Primer specificity was tested by PCR using plasmids containing each of the six B. napus PSY genes. Primers BnaC.PSY.a and BnaA.PSY.b could not be tested against BnaA.PSY.d and BnaC.PSY.f (clones did not include 5′UTRs). B. SSCP analysis of BnaC.PSY.a RT-PCR reactions show that only two strands exhibiting the same exact pattern as the BnaC.PSY.a plasmid control are present, confirming primer specificity. C. SSCP analysis of BnaA.PSY.b RT-PCR reactions show that only two strands exhibiting the same exact pattern as the BnaA.PSY.b plasmid control are present, confirming primer specificity. L: 100 bp ladder; BnaX.PSY.a-f: plasmid DNA controls; gDNA: B. napus genomic DNA control; WC: water control; ND. Not determined; 20 dpa: seed cDNA 20 days post anthesis; Leaf: leaf cDNA. S4 Figure. Alignment of B. napus PSY proteins with squalene synthase and carotenoid dehydrosqualene synthase templates. 2zcs: Staphylococcus aureus Dehydrosqualene synthase complexed with BPH-700; 3acx: Staphylococcus aureus Dehydrosqualene synthase complexed with BPH-673; 4e9u: Staphylococcus aureus Dehydrosqualene synthase complexed with thiocyanate inhibitor; 2zco: Staphylococcus aureus Dehydrosqualene synthase; 4hdl: PpcA F15L mutant from Geobacter sulfurreducens; 3vj8: Homo sapiens Squalene synthase. S5 Figure. Tridimensiona [file pone.0114878.s001.docx]

**Supporting Information File 1:**


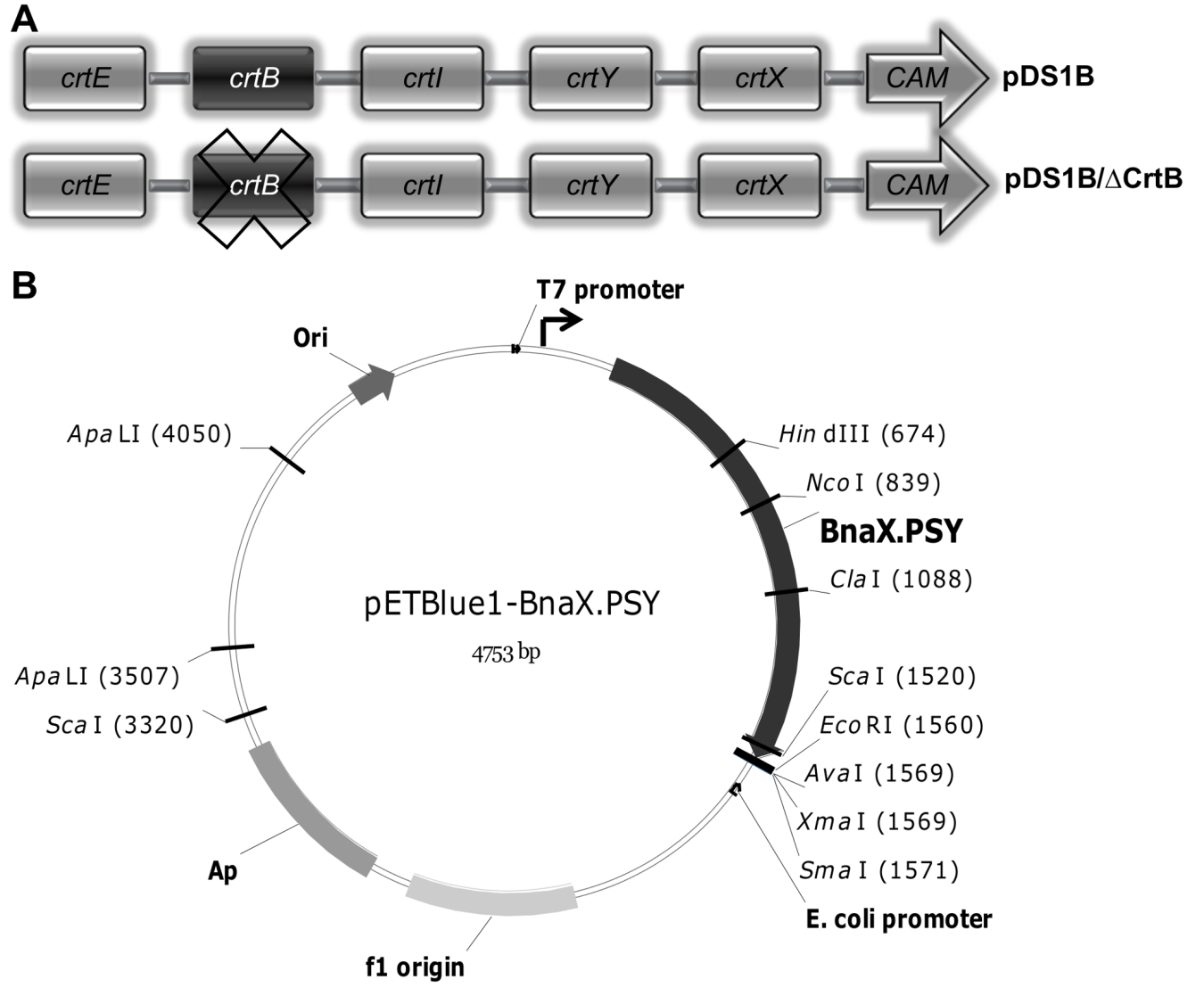


**Figure S1.** **PSY Heterologous Complementation System.** A. Two *E. coli* BL21-Gold strains were used, a β-carotene producer strain (DS1B) transformed with plasmid pDS1B, a pBAD33 vector carrying *Erwinia* *uredovora* carotenogenic genes *crtE*, *crtB*, *crtI*, *crtY* and *CrtX* and a non-producer strain (DS1B-Δ*crtB*) transformed with plasmid pDS1B-Δ*crtB*, which has a deletion of the *Eu crtB* gene. B. Six pETBlue1-BnaX.PSY vectors were used, each carrying a *B. napus* PSY homologue, without its corresponding signal peptide, cloned into the EcoRV site.

10 20 30 40 50 60 70 80 90 100

....|....|....|....|....|....|....|....|....|....|....|....|....|....|....|....|....|....|....|....|

**BnaC.PSY.a** **ATGTCTTCT---GTAGCAGTGTTATGGGTTGCTTCCTCTTCTCCTAATCCAGACCCGATGAACACTTGTGGGTTGGTAAGGGCTGTAGAGTCTTCTAGAG**

**BnaA.PSY.b** **ATGTCTTCT---GTAGCAGTGTTATGGGTTGCTTCCTCTTCTCCAAATCCAGACCCGATGAACACTTGTGGGTTGGTAAGGGCTGTAGAATCTTCTAGAG**

**BnaA.PSY.c** **ATGTCGTCT---GTAGTAGTGTTATGGGTTGCT---CCTTCTCCAAATCCAGACCCAATGAACACTTGTGGGTTGGTAAGGGCTTTAGAATCTTCTAGAG**

**BnaA.PSY.d** **ATGTCTTCT---GTAGCAGTGTTATGGGTTGCTCCCTCTTCTCCAAATCCAGACCCAATGACCAATTCTGGGTTGGTAAGGGTTCTAGAGTCTTCTAGAT**

**BnaC.PSY.e** **ATGTCTTCT---GTAGCAGTGTTATGGGTTGCTGCTTCTTCTCCAAATCCAGACCCAATGAACACTTGTGGGTTGGTACGGGCTTTAGAATCTTCTAGAG**

**BnaC.PSY.f** **ATGTCTTCT---GTAGCAGTGTTATGGGTTGCTCCCTCTTCTCCAATTCCAGACCCAATGAACAATCCTGGGTTAATAAGAGTTCTAGAATCTTCTAGAT**

**AtPSY**  **ATGTCTTCTTCTGTAGCAGTGTTATGGGTTGCTACTTCTTCTCTAAATCCAGACCCAATGAACAATTGTGGGTTGGTAAGGGTTCTAGAATCTTCTAGAC**

**Consensus**  **ATGTCTTCT---GTAGCAGTGTTATGGGTTGCTTCCTCTTCTCCTAATCCAGACCCGATGAACACTTGTGGGTTGGTAAGGGCTGTAGAGTCTTCTAGAG**

110 120 130 140 150 160 170 180 190 200

....|....|....|....|....|....|....|....|....|....|....|....|....|....|....|....|....|....|....|....|

**BnaC.PSY.a** **TTCTCTCTCGTTGTCAGAATCAGAGAATGGACAATGGTAGGAGGAAGCAAACAACA---AAAACGTGGACCTCTTCTTCTTCTTCCTCTCTAATGAGCT-**

**BnaA.PSY.b** **TGCTCTCTCGTTGTCAGAATCAGAGAATGGACAATGGTAGGAGGAAGCAAACAACA---AAAACATGGACTTCTTCCTCTTCTTC---TGTAATGAGCT-**

**BnaA.PSY.c** **TGCTCTCTCGTTGTCAGA------GAATGAGCAATGGTAGGAGGAAGCAAACAACA---ACAACTTGGAGTTCTTCT------------GTTATGAGAC-**

**BnaA.PSY.d** **TGCTTTCTTCTTGTCTGAATCAGAGACTCAACACTGGCAAGAGGA------------------------GTTCTTCTTCTCCTTCTTCTCTAGTGAGCTG**

**BnaC.PSY.e** **TGCTCTCTCGTTGTCAGAATCAGAGAGTGAACAATGGTAGGAGGAACCAAACAACACAAACTACTTGGAGTTCTTCTTCTTCT------GTAATGAGCTT**

**BnaC.PSY.f** **TGCTTTCTCCTTGTCTGAATCAGAGACTCGACACTGGCAAGAGGA------------------------GTTCTTCTT------CTTCTGTAATGAGCTG**

**AtPSY**  **TGTTCTCTCCTTGTCAGAATCAGAGACTAAACAAAGGTAAGAAGAAGCAGATACCA------ACTTGGAGTTCTTCTTTT---------GTAAGGAACC-**

**Consensus**  **TTCTCTCTCGTTGTCAGAATCAGAGAATGGACAATGGTAGGAGGAAGCAAACAACA---AAAACGTGGACCTCTTCTTCTTCTTCCTCTCTAATGAGCT-**

210 220 230 240 250 260 270 280 290 300

....|....|....|....|....|....|....|....|....|....|....|....|....|....|....|....|....|....|....|....|

**BnaC.PSY.a** **--------ACAGAAGGAATGTTGTGTCTTCGAGCGTAGTAGCAAGTCATGC---AGGAGAGATAGCACTCTCATCTGAAGAGAAGGTTTACAACGTTGTG**

**BnaA.PSY.b** **--------ACAGAAGGAGTGTGGTGTCTTCGAGCGTAGTAGCAAGTCATGC---AGGAGAGATAGCACTCTCTTCTGAAGAGAAGGTTTACAACGTTGTG**

**BnaA.PSY.c** **--GCCGCAGAAGAAGTAGTGTTGTGTCTTCAAGCTTAGTCGTAAATCCTGC---AGGAGAGATGACCCTCTCATCTGAAGAGAAGGTTTACAATGTTGTG**

**BnaA.PSY.d** **CAGAAGGGGAAGAAGTAGTGTTGTGTCTTCAAGCTTAGTAGCAAGTCCTGCTGCAGGAGAGATAACCCTCTCATCTGAAGAGAAGGTTTACAACGTTGTG**

**BnaC.PSY.e** **TAGAAGAAGAAGAAGCAGTGTTGTCTCTTCAAGCTTAGTAGCACATCCTGC---AGGAGAGATAGACCTCTCATCTGAAGAGAAGGTTTACAATGTTGTG**

**BnaC.PSY.f** **CAGAAGAAGATTAAGTAGTGCTGTCTCTTCAAGTTTAGTAGCAAGTCCTGCTGCAGGAGAGATAACCCTCTCATCTGAAGAGAAGGTTTACAACGTTGTG**

**AtPSY**  **--GAAGTAGAAGAATTGGTGTTGTGTCTTCAAGCTTAGTAGCAAGTCCTTCT---GGAGAGATAGCTCTTTCATCTGAAGAGAAGGTTTACAATGTTGTG**

**Consensus**  **--------ACAGAAGGAATGTTGTGTCTTCGAGCGTAGTAGCAAGTCATGC---AGGAGAGATAGCACTCTCATCTGAAGAGAAGGTTTACAACGTTGTG**

310 320 330 340 350 360 370 380 390 400

....|....|....|....|....|....|....|....|....|....|....|....|....|....|....|....|....|....|....|....|

**BnaC.PSY.a** **CTTAGACAAGCGGCTTTGGTTAACAAACAGCTCAGGTCTACTTCTCCTGAACTTGATGATGTGAAGAAACCACGGGATATTGTTCTCCCTGGGA------**

**BnaA.PSY.b** **CTGAGACAAGCGGCTTTGGTGAACAAACAGCTCAGGTCTACTTCTCCTGAACTTGATGATGTGAAGAAACCACAGGATATTGTTCTCCCTGGGA------**

**BnaA.PSY.c** **CTGAGTCAAGCAGCTTTGGTGAACGAACAGCTACGGTCTACTTCTCCTGTTCTTGATGATGTGAAGAAACCACAGGATACTGTTCGTCCTGGGA------**

**BnaA.PSY.d** **TTGAAACAAGCAGCTTTGGGTAACAAACAGCTTAGG------------GACCTTGATGATGTGAAGAAGCCACAAGATATTGTTCTTCCTGGGACTACTA**

**BnaC.PSY.e** **TTGAGACAAGCAGCTTTGGTGAACAAACAGCTTAGGTCTACTTCTCCTGTTCTTGACGATGTGAAGAAACCACAGGATATTGTTCGTCCTGGGA------**

**BnaC.PSY.f** **CTGAAACAAGCAGCTCTGGTTAACAAACAGCTTAGG------------GACCTTGATGATCTGAAGAAGCCACAGGATATTGTTCTTCCTGGGACTACTA**

**AtPSY**  **TTGAAACAAGCTGCTTTGGTGAACAAACAGCTAAGGTCTTCTTCTTATGACCTTGATG---TGAAGAAACCACAAGATGTTGTTCTTCCTGGGA------**

**Consensus**  **CTTAGACAAGCGGCTTTGGTTAACAAACAGCTCAGGTCTACTTCTCCTGAACTTGATGATGTGAAGAAACCACGGGATATTGTTCTCCCTGGGA------**

410 420 430 440 450 460 470 480 490 500

....|....|....|....|....|....|....|....|....|....|....|....|....|....|....|....|....|....|....|....|

**BnaC.PSY.a** **------GTTTGAGTTTGTTGGGTGAAGCTTATGATCGTTGCGGCGAAGTTTGCGCTGAATATGCTAAAACCTTTTATCTTGGAACTTTGCTTATGACACC**

**BnaA.PSY.b** **------GTTTGAGTTTGTTGGGTGAAGCTTATGATCGTTGCGGCGAAGTTTGCGCTGAATATGCTAAAACCTTTTATCTTGGAACTTTGCTTATGACACC**

**BnaA.PSY.c** **------GTTTGAGTTTGTTGGGTGAAGCTTATGATCGATGTGGCGAAGTTTGTGCTGAATATGCTAAAACGTTTTATCTTGGAACTTTGCTTATGACACC**

**BnaA.PSY.d** **CTGGGAGTTTGAGTTTGTTGGGTGAAGCTTACGATCGTTGCGGTGAAGTTTGCGCTGAATATGCCAAAACGTTTTATCTCGGAACTTTGCTTATGACACC**

**BnaC.PSY.e** **------GTTTGAGCTTGTTGGCTGAAGCTTATGATCGATGCGGCGAAGTTTGTGCTGAATATGCCAAAACGTTTTATCTTGGAACTTTGCTTATGACACC**

**BnaC.PSY.f** **CTGGGAGTTTGAGTTTGTTGGGTGAAGCTTATGATCGATGCGGCGAAGTTTGTGCTGAATATGCTAAAACGTTTTATCTCGGAACTTTGCTTATGACACC**

**AtPSY**  **------GTTTGAGTTTGTTGGGTGAAGCTTATGATCGATGCGGTGAAGTTTGCGCTGAATATGCTAAGACGTTTTATCTTGGAACTTTGCTTATGACACC**

**Consensus**  **------GTTTGAGTTTGTTGGGTGAAGCTTATGATCGTTGCGGCGAAGTTTGCGCTGAATATGCTAAAACCTTTTATCTTGGAACTTTGCTTATGACACC**

510 520 530 540 550 560 570 580 590 600

....|....|....|....|....|....|....|....|....|....|....|....|....|....|....|....|....|....|....|....|

**BnaC.PSY.a** **AGAGAGGCGAAAGGCGATTTGGGCTATATA---------------------------------------------CGTTTGGTGCAGAAGAACTGATGAG**

**BnaA.PSY.b** **TGAGAGGCGAAAGGCCATTTGGGCTATCTA---------------------------------------------CGTTTGGTGCAGAAGAACTGATGAG**

**BnaA.PSY.c** **TGAAAGGCGCAAAGCTATTTGGGCTATCTA---------------------------------------------CGTGTGGTGTAGAAGAACTGATGAA**

**BnaA.PSY.d** **TGAGAGGCGAAAGGCCATTTGGGCTATCTA---------------------------------------------TGTTTGGTGTAGAAGAACTGATGAG**

**BnaC.PSY.e** **CGAGAGGAGAAAGGCGATTTGGGCTATCTA---------------------------------------------TGTTTGGTGCAGAAGAACTGATGAA**

**BnaC.PSY.f** **TGAGAGGCGAAAGGCCATTTGGGCTATCTA---------------------------------------------TGTTTGGTGTAGAAGAACTGATGAA**

**AtPSY**  **CGAAAGGCGAAAGGCGATTTGGGCAATCTACGTGATGCTCAAAGTAGATTTCTACAAACAATCTATTGTGGCTCTTGTTTGGTGTAGAAGAACTGATGAA**

**Consensus**  **AGAGAGGCGAAAGGCGATTTGGGCTATATA---------------------------------------------CGTTTGGTGCAGAAGAACTGATGAG**

610 620 630 640 650 660 670 680 690 700

....|....|....|....|....|....|....|....|....|....|....|....|....|....|....|....|....|....|....|....|

**BnaC.PSY.a** **CTGGTAGATGGGCCTAATGCATCACACATAACTCCCATGGCGTTAGATAGATGGGAAGCAAGGCTAGAAGATCTATTCCGTGGCCGTCCATTCGATATGC**

**BnaA.PSY.b** **CTGGTAGATGGGCCTAATGCATCACATATTACTCCCATGGCGTTAGATAGATGGGAAGCAAGGCTAGAAGATCTATTCCGTGGCCGTCCTTTCGATATGC**

**BnaA.PSY.c** **CTAGTAGATGGGCCTAATGCATCACATATAACTCCCATGGCGTTAGATAGATGGGAAGCAAGGTTAGAAGATCTTTACCGTGGCCGTGCTTTCGATATGC**

**BnaA.PSY.d** **CTCGTAGATGGGCCTAATGCATCACACATAACTCCCATGGCGTTAGATAGATGGGAAGCAAGGTTAGAAGATCTCTTCCGTGGCCGTCCTTTCGATATGC**

**BnaC.PSY.e** **CTGGTAGATGGGCCGAATGCATCACATATAACTCCCATGGCGTTAGATAGATGGGAAGCAAGGTTAGAAGATCTTTTTCATGGCCGTGCTTTCGATATGC**

**BnaC.PSY.f** **CTCGTAGATGGGCCTAATGCATCACACATAACACCCATGGCGTTAGATAGATGGGAAGCAAGGTTAGAAGATCTTTTCCGTGGCCGTCCTTTCGATATGC**

**AtPSY**  **CTTGTGGATGGGCCAAATGCTTCACATATAACTCCCATGGCTTTAGATAGATGGGAAGCAAGGTTAGAAGATCTTTTCCGTGGTCGTCCTTTCGATATGC**

**Consensus**  **CTGGTAGATGGGCCTAATGCATCACACATAACTCCCATGGCGTTAGATAGATGGGAAGCAAGGCTAGAAGATCTATTCCGTGGCCGTCCATTCGATATGC**

710 720 730 740 750 760 770 780 790 800

....|....|....|....|....|....|....|....|....|....|....|....|....|....|....|....|....|....|....|....|

**BnaC.PSY.a** **TTGATGCTGCTCTCGCTGATACCGTTGCTAGATACCCTGTCGATATTCAGCCATTTAGAGACATGATCGAAGGAATGAGAATGGATTTGAGGAAGTCCAG**

**BnaA.PSY.b** **TTGATGCTGCTCTCGCTGATACTGTTGCTAGATACCCTGTTGATATTCAGCCATTTAGAGACATGATCGAAGGAATGAGAATGGATTTGAGGAAGTCCAG**

**BnaA.PSY.c** **TTGACGCTGCCCTCGCTGATACAGTTACTAGATACCCTGTAGATATTCAGCCATTTAGAGACATGATCGAAGGAATGAGAATGGATTTGAGGAAGTCTAG**

**BnaA.PSY.d** **TTGACGCTGCTCTAGCTGATACAGTTGCTAGATACCCTGTAGATATTCAGCCATTTAGAGACATGGTAGAAGGAATGAGAATGGATCTGAGGAAGTCTAG**

**BnaC.PSY.e** **TTGACGCTGCTCTAGCTGATACAGTTACTAGATACCCTGTAGATATTCAGCCATTTAGAGACATGATTGAAGGAATGAGAATGGATTTGAGGAAGTCTAG**

**BnaC.PSY.f** **TTGACGCTGCTCTAGCTGATACAGTTGCTAGATACCCCGTCGATGTTCAGCCATTTAGAGACATGGTAGAAGGAATGAGAATGGATCTGAGGAAGTCTAG**

**AtPSY**  **TTGATGCTGCTCTCGCTGATACAGTTGCTAGATACCCGGTCGATATTCAGCCATTTCGAGACATGATCGAAGGAATGAGAATGGACTTGAAGAAATCGAG**

**Consensus**  **TTGATGCTGCTCTCGCTGATACCGTTGCTAGATACCCTGTCGATATTCAGCCATTTAGAGACATGATCGAAGGAATGAGAATGGATTTGAGGAAGTCCAG**

810 820 830 840 850 860 870 880 890 900

....|....|....|....|....|....|....|....|....|....|....|....|....|....|....|....|....|....|....|....|

**BnaC.PSY.a** **ATACAAGAACTTTGATGATCTCTACCTTTACTGCTACTATGTAGCCGGAACCGTCGGTTTGATGAGCGTTCCGGTTATGGGAATCGATCCCAAGTCCAAA**

**BnaA.PSY.b** **ATACAAGAACTTCGATGATCTCTACCTTTACTGCTACTATGTAGCTGGAACCGTCGGTTTGATGAGCGTTCCGGTTATGGGAATCGATCCTAAATCCAAA**

**BnaA.PSY.c** **ATACAACAACTTTGATGAGCTCTACCTTTACTGCTACTATGTGGCGGGAACCGTCGGTTTGATGAGCGTTCCGGTTATGGGAATCGATCCCAAGTCCAAA**

**BnaA.PSY.d** **ATACAAGAACTTTGATGATCTCTACCTTTACTGCTACTATGTAGCTGGAACCGTAGGTTTGATGAGCGTTCCGGTTATGGGGATCGATCCTAAGTCCAAA**

**BnaC.PSY.e** **ATACAACAACTTTGATGAGCTCTACCTTTACTGCTACTATGTCGCCGGAACCGTCGGTTTGATGAGCGTTCCGGTTATGGGAATCAATCCCAAGTCCAAG**

**BnaC.PSY.f** **ATACAAGAACTTTGATGATCTCTACCTTTACTGCTACTATGTAGCCGGAACCGTCGGTTTGATGAGCGTTCCGGTTATGGGGATCGATCCCAAGTCCAAA**

**AtPSY**  **ATACCAGAACTTCGATGATCTATACCTTTACTGCTACTACGTCGCTGGAACCGTCGGATTGATGAGCGTTCCGGTTATGGGAATCGATCCTAAGTCGAAA**

**Consensus**  **ATACAAGAACTTTGATGATCTCTACCTTTACTGCTACTATGTAGCCGGAACCGTCGGTTTGATGAGCGTTCCGGTTATGGGAATCGATCCCAAGTCCAAA**

910 920 930 940 950 960 970 980 990 1000

....|....|....|....|....|....|....|....|....|....|....|....|....|....|....|....|....|....|....|....|

**BnaC.PSY.a** **GCAACGACCGAGAGTGTTTACAACGCTGCCTTGGCTCTCGGTATAGCTAATCAGCTTACCAACATACTCAGAGACGTTGGCGAAGATGCAAGAAGAGGAA**

**BnaA.PSY.b** **GCAACGACCGAGAGTGTTTACAACGCTGCCTTGGCTCTCGGTATAGCTAATCAGCTTACCAACATACTCAGAGACGTTGGCGAAGATGCGAGAAGAGGAA**

**BnaA.PSY.c** **GCAACGACCGAGAGTGTTTACAACGCTGCCTTGGCCCTCGGTATAGCCAATCAGCTTACCAACATACTCAGAGACGTTGGTGAAGATGCAAGAAGAGGAA**

**BnaA.PSY.d** **GCAACGACCGAGAGTGTTTACAACGCTGCCTTGGCCCTTGGTATAGCTAATCAGCTTACCAACATACTCAGAGACGTTGGCGAAGATGCGAGAAGAGGAA**

**BnaC.PSY.e** **GCAACGACCGAGAGTGTTTACAACGCTGCCTTGGCCCTCGGTATAGCCAATCAGCTTACCAACATACTCAGGGACGTTGGTGAAGATGCAAGAAGAGGAA**

**BnaC.PSY.f** **GCAACGACAGAGAGTGTTTACAACGCTGCCTTGGCTCTCGGTATAGCCAATCAGCTTACCAACATACTCAGAGACGTTGGCGAAGATGCGAGAAGAGGAA**

**AtPSY**  **GCAACAACCGAAAGTGTTTACAACGCTGCCTTGGCCCTTGGTATAGCCAATCAGCTTACTAACATACTCAGAGACGTAGGCGAAGATGCGAGAAGAGGAA**

**Consensus**  **GCAACGACCGAGAGTGTTTACAACGCTGCCTTGGCTCTCGGTATAGCTAATCAGCTTACCAACATACTCAGAGACGTTGGCGAAGATGCAAGAAGAGGAA**

1010 1020 1030 1040 1050 1060 1070 1080 1090 1100

....|....|....|....|....|....|....|....|....|....|....|....|....|....|....|....|....|....|....|....|

**BnaC.PSY.a** **GAGTTTATCTGCCCCAAGATGAGTTAGCTCAAGCTGGTCTCTCAGATGAAGACATATTCGCGGGAAAAGTCACTGATAAATGGAGGAACTTCATGAAAAT**

**BnaA.PSY.b** **GAGTTTATCTACCGCAAGATGAGTTAGCTCAAGCTGGTCTCTCAGATGAAGACATCTTCGCGGGAAAAGTCACTGACAAATGGAGAAACTTCATGAAAAT**

**BnaA.PSY.c** **GAGTTTATCTACCACAAGATGAGTTAGCTCAAGCTGGTCTCTCAGATGAAGATATATTTGCTGGAAAAGTCACTGATAAATGGAGAAACTTCATGAGAAT**

**BnaA.PSY.d** **GGGTTTATCTGCCCCAAGATGAACTAGCTCAGGCTGGTCTCTCAGATGAAGACATATTCGCTGGAAAAGTCACTGATAAATGGAGAAACTTCATGAAAAT**

**BnaC.PSY.e** **GAGTTTATCTACCACAAGATGAGTTAGTTCAGGCTGGTCTCTCCGATGAAGACATATTCGCTGGAAAAGTCACTGATAAATGGAGAAACTTCATGAGACT**

**BnaC.PSY.f** **GGGTTTATCTGCCACAAGATGAGCTAGCTCAGGCTGGTCTCTCAGATGAAGACATATTCGCTGGAAAAGTCACTGATAAATGGAGAAACTTCATGAAAAT**

**AtPSY**  **GGGTTTATCTGCCTCAGGATGAATTGGCTCAGGCTGGTCTTTCAGATGAAGACATATTCGCCGGAAAAGTAACTGATAAATGGAGAAACTTCATGAAAAT**

**Consensus**  **GAGTTTATCTGCCCCAAGATGAGTTAGCTCAAGCTGGTCTCTCAGATGAAGACATATTCGCGGGAAAAGTCACTGATAAATGGAGGAACTTCATGAAAAT**

1110 1120 1130 1140 1150 1160 1170 1180 1190 1200

....|....|....|....|....|....|....|....|....|....|....|....|....|....|....|....|....|....|....|....|

**BnaC.PSY.a** **GCAGCTTAAGCGAGCAAGAATGTTCTTTGACGAAGCTGAGAAAGGCATTACTGAGCTGGACGCTGCTAGCAGATGGCCGGTATGGGCGTCGCTCCTATTG**

**BnaA.PSY.b** **GCAGCTTAAGCGAGCAAGAATGTTCTTTGACGAAGCTGAGAAAGGTGTTACAGAGCTCGACGCTGCTAGCAGATGGCCGGTGTGGGCATCTCTCCTATTG**

**BnaA.PSY.c** **GCAGCTTAAGAGAGCAAGAATGTTCTTTGACGAAGCTGAGAAAGGTGTCACCGAGCTTGACGCTGCTAGCAGATGGCCAGTATGGGCATCGCTCCTATTA**

**BnaA.PSY.d** **GCAGCTTAAGCGAGCAAGAATGTTCTTTGATGAAGCTGAGAAAGGTGTTACTGAGCTAGACGCAGCTAGCAGATGGCCGGTATGGGCATCGCTCCTATTG**

**BnaC.PSY.e** **GCAGCTTAAGAGAGCAAGAATGTTCTTCGACGAAGCTGAGAAAGGTGTCACCGAGCTTGACGCTGCTAGCAGATGGCCAGTATGGGCATCGCTACTATTG**

**BnaC.PSY.f** **GCAGCTTAAGCGAGCAAGAATGTTCTTTGATGAAGCTGAGAAAGGTGTTACTGAGCTTGACGCAGCTAGCAGATGGCCGGTATGGGCATCGCTCCTATTG**

**AtPSY**  **GCAGCTTAAACGAGCAAGAATGTTCTTCGACGAAGCTGAGAAAGGCGTCACCGAGCTCAGTGCCGCTAGCAGATGGCCTGTATGGGCTTCATTGCTATTG**

**Consensus**  **GCAGCTTAAGCGAGCAAGAATGTTCTTTGACGAAGCTGAGAAAGGCATTACTGAGCTGGACGCTGCTAGCAGATGGCCGGTATGGGCGTCGCTCCTATTG**

1210 1220 1230 1240 1250 1260 1270 1280 1290 1300

....|....|....|....|....|....|....|....|....|....|....|....|....|....|....|....|....|....|....|....|

**BnaC.PSY.a** **TACAGGAGAATATTGGACGAGATTGAAGCGAATGATTACAACAACTTTACGAAGAGAGCTTATGTGGGGAAAGCCAAGAAAATTGCAGCTCTGCCATTGG**

**BnaA.PSY.b** **TACAGGAGAATATTGGACGAGATTGAAGCGAATGATTACAACAACTTTACGAAGAGAGCTTATGTTGGGAAAGCCAAGAAAATTGCAGCTCTGCCATTGG**

**BnaA.PSY.c** **TACAGGAGAATATTAGACGAGATTGAAGCGAATGATTACAACAACTTTACAAAGAGAGCTTATGTTGGGAAAGTGAAGAAAATTGCAGCTCTTCCATTAG**

**BnaA.PSY.d** **TACAGGAGGATATTGGACGAGATTGAAGCGAATGATTACAACAACTTTACAAAGAGAGCTTATGTTGGGAAAGCCAAGAAGATTGCAGCTCTGCCATTAG**

**BnaC.PSY.e** **TATAGGAGAATATTGGACGAGATTGAAGCGAATGATTACAACAACTTTACCAAGAGAGCTTATGTTGGGAAAGTTAAGAAAATTGCAGCTCTTCCATTAG**

**BnaC.PSY.f** **TACAGGAGGATATTGGACGAGATTGAAGCGAATGATTGCAACAACTTTACGAAGAGAGCTTATGTTGGGAAAGCGAAGAAGATTGCAGCTCTCCCATTAG**

**AtPSY**  **TACAGGAGAATACTGGACGAGATTGAAGCGAATGATTACAACAATTTTACTAAGAGAGCTTATGTGGGGAAAGTCAAGAAAATTGCAGCTTTGCCATTGG**

**Consensus**  **TACAGGAGAATATTGGACGAGATTGAAGCGAATGATTACAACAACTTTACGAAGAGAGCTTATGTGGGGAAAGCCAAGAAAATTGCAGCTCTGCCATTGG**

1310 1320 1330 1340 1350

....|....|....|....|....|....|....|....|....|....|

**BnaC.PSY.a** **CTTATGCTAAATCAGTACTAAAGACTCCAAGTTCAAGAGGAACAACTTAA**

**BnaA.PSY.b** **CTTATGCTAAATCAGTACTAAAGACTCCAAGTTCAAGAGGAACAACTTAA**

**BnaA.PSY.c** **CTTATGCTAAATCAGTACTAAAGGTTTCAAGTTAA---------------**

**BnaA.PSY.d** **CTTATGCTAAATCAATACTAAAGGCTTCAAGTTCAAGATGA---------**

**BnaC.PSY.e** **CTTATGCTAAATCAGTAGTAAAGGTTTCAAGTTAA---------------**

**BnaC.PSY.f** **CTTATGCTAAATCAATACTAAAGGCTTCAAGTTCAAGATGA---------**

**AtPSY**  **CTTATGCTAAATCAGTACTAAAGACTTCAAGTTCAAGACTATCGATATGA**

**Consensus**  **CTTATGCTAAATCAGTACTAAAGACTCCAAGTTCAAGAGGAACAACTTAA**

**Figure S2. Multiple nucleotide *BnaX.PSY* sequence alignment.**


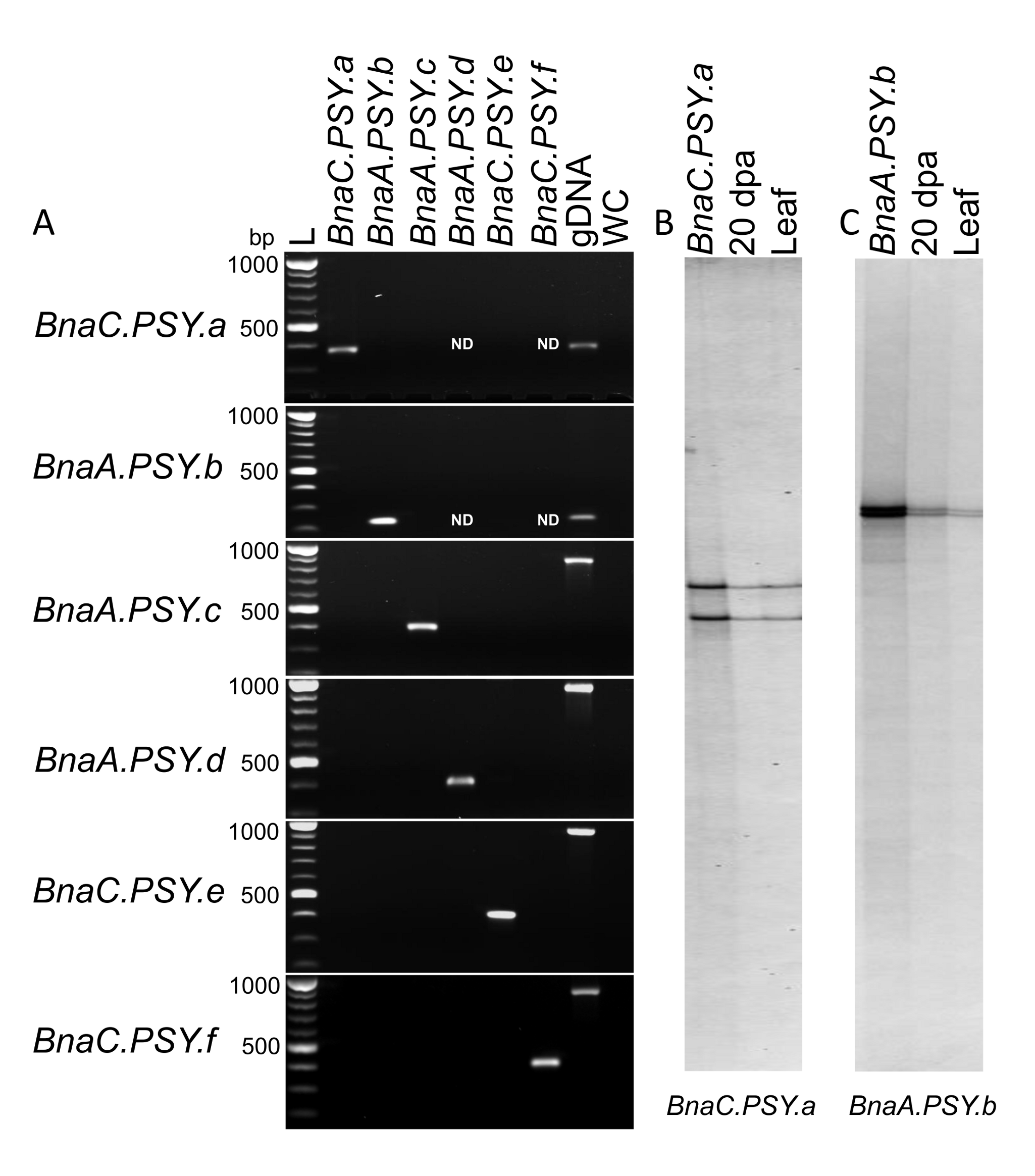


**Figure S3. Homologue-specific PCR primer control reactions. A.** Primer specificity was tested by PCR using plasmids containing each of the six *B. napus* PSY genes. Primers BnaC.PSY.a and BnaA.PSY.b could not be tested against BnaA.PSY.d and BnaC.PSY.f (clones did not include 5’UTRs). **B.** SSCP analysis of *BnaC. PSY.a* RT-PCR reactions show that only two strands exhibiting the same exact pattern as the *BnaC.PSY.a* plasmid control are present, confirming primer specificity. **C.** SSCP analysis of *BnaA. PSY.b* RT-PCR reactions show that only two strands exhibiting the same exact pattern as the *BnaC.PSY.b* plasmid control are present, confirming primer specificity. L: 100 bp ladder; BnaX.PSY.a-f: plasmid DNA controls; gDNA: *B*. *napus* genomic DNA control; WC: water control; ND. Not determined; 20 dpa: seed cDNA 20 days post anthesis; Leaf: leaf cDNA.

**BnaC.PSY.a**

PSY_A/1-349 ASHAGEIALSSEEKVYNVVLRQAALVNKQLRSTSPELDDVKKPRDIVLPGSLSLLGEAYD

2zcsA/1-281 ---------------------------------------------------MTMMDMNFK

3acxA/1-277 ---------------------------------------------------MTMMDMNFK

4e9uA/1-281 ---------------------------------------------------MTMMDMNFK

2zcoA/1-281 MT---------------------------------------------------MMDMNFK

2zcsA/1-281 ---------------------------------------------------MTMMDMNFK

3acxA/1-277 ---------------------------------------------------MTMMDMNFK

4r9uA/1-281 ---------------------------------------------------MTMMDMNFK

2zcsA/1-275 ---------------------------------------------------------NFK

4hdlA/1-258 ----------------------------------------------NAGSVPVELRGDFE

3vj8A/1-293 -----------------------------------------------------SLSSSLK

2nd_struc_prediction/1-349 CCCCCCCCCCCCCCHHHHHHHHHHHHHHHHCCCCCCCCCCCCCCCCCCCCCCCCCHHHHH

PSY_A/1-349 RCGEVCAEYAKTFYLGTLLMTPERRKAIWAIYVWCRRTDELVDGPNASHITPMALDRWEA

2zcsA/1-281 YCHKIMKKHSKSFSYAFDLLPEDQRKAVWAIYAVCRKIDDSIDVYGDINQIKEDIQSIEK

3acxA/1-277 YCHKIMKKHSKSFSYAFDLLPEDQRKAVWAIYAVCRKIDDSIDVYGDIQF----LNQIKE

4e9uA/1-281 YCHKIMKKHSKSFSYAFDLLPEDQRKAVWAIYAVCRKIDDSIDVYGDINQIKEDIQSIEK

2zcoA/1-281 YCHKIMKKHSKSFSYAFDLLPEDQRKAVWAIYAVCRKIDDSIDVYGDIQQIKEDIQSIEK

2zcsA/1-281 YCHKIMKKHSKSFSYAFDLLPEDQRKAVWAIYAVCRKIDDSIDVYGDINQIKEDIQSIEK

3acxA/1-277 YCHKIMKKHSKSFSYAFDLLPEDQRKAVWAIYAVCRKIDDSIDVYGDIQF----LNQIKE

4r9uA/1-281 YCHKIMKKHSKSFSYAFDLLPEDQRKAVWAIYAVCRKIDDSIDVYGDINQIKEDIQSIEK

2zcsA/1-275 YCHKIMKKHSKSFSYAFDLLPEDQRKAVWAIYAVCRKIDDSIDVYGDIQFLNQIKEDIQS

4hdlA/1-258 VCRRLTRSHYENFSVVSLFVPRHLRPHFYSVYAFCRGVDDLGDEFAGD---RAALDAYEE

3vj8A/1-293 TCYKYLNQTSRSFAAVIQALDGEMRNAVCIFYLVLRALDTLEDDMTSVEKKVPLLHNFHS

2nd_struc_prediction/1-349 HHHHHHHCCCCCHHHHHHHCCHHHHHHHHHHHHHHHHHHHHHCCCCCCHHHHHHHHHHHH

PSY_A/1-349 RLEDLFRGRPFDMLDAALADTVARYPVDIQPFRDMIEGMRMDLRKSRYKNFDDLYLYCYY

2zcsA/1-281 YPYEYHHFQSDRRIMMALQHVAQHKNIAFQSFYNLIDTVYKDQHFTMFETDAELFGYCYG

3acxA/1-277 DIQSIEKYPSDRRIMMALQHVAQHKNIAFQSFYNLIDTVYKDQHFTMFETDAELFGYCYG

4e9uA/1-281 YPYEYHHFQSDRRIMMALQHVAQHKNIAFQSFYNLIDTVYKDQHFTMFETDAELFGYCYG

2zcoA/1-281 YPYEYHHFQSDRRIMMALQHVAQHKNIAFQSFYNLIDTVYKDQHFTMFETDAELFGYCYG

2zcsA/1-281 YPYEYHHFQSDRRIMMALQHVAQHKNIAFQSFYNLIDTVYKDQHFTMFETDAELFGYCYG

3acxA/1-277 DIQSIEKYPSDRRIMMALQHVAQHKNIAFQSFYNLIDTVYKDQHFTMFETDAELFGYCYG

4r9uA/1-281 YPYEYHHFQSDRRIMMALQHVAQHKNIAFQSFYNLIDTVYKDQHFTMFETDAELFGYCYG

2zcsA/1-275 IEKYPYEYQSDRRIMMALQHVAQHKNIAFQSFYNLIDTVYKDQHFTMFETDAELFGYCYG

4hdlA/1-258 ELRRAFAGEATTPAFRALQFTIATCNLP-EPFLRLIEANRRDQRKHTYDTWEDLRDYCRY

3vj8A/1-293 FLYQRFMESKDRQVLEDFPTISLEFRNLAEKYQTVIIGMAEFLD-KHVTSEQEWDKYCHY

2nd_struc_prediction/1-349 HHHHHHCCCCCCHHHHHHHHHHHHHCCCHHHHHHHHHHHHHHHCCCCCCCHHHHHHHHHH

PSY_A/1-349 VAGTVGLMSVPVMGIDPKSKATTESVYNAALALGIANQLTNILRDVGEDARRGRVYLPQD

2zcsA/1-281 VAGTVGEVLTPILSDHET-----HQTYDVARRLGESLQLINILRDVGEDFENERIYFSKQ

3acxA/1-277 VAGTVGEVLTPILSDHE-----THQTYDVARRLGESLQLINILRDVGEDFENERIYFSKQ

4e9uA/1-281 VAGTVGEVLTPILSDHET-----HQTYDVARRLGESLQLINILRDVGEDFENERIYFSKQ

2zcoA/1-281 VAGTVGEVLTPILSDHET-----HQTYDVARRLGESLQLINILRDVGEDFENERIYFSKQ

2zcsA/1-281 VAGTVGEVLTPILSDHET-----HQTYDVARRLGESLQLINILRDVGEDFENERIYFSKQ

3acxA/1-277 VAGTVGEVLTPILSDHE-----THQTYDVARRLGESLQLINILRDVGEDFENERIYFSKQ

4r9uA/1-281 VAGTVGEVLTPILSDHET-----HQTYDVARRLGESLQLINILRDVGEDFENERIYFSKQ

2zcsA/1-275 VAGTVGEVLTPILSDHET-----HQTYDVARRLGESLQLINILRDVGEDFENERIYFSKQ

4hdlA/1-258 SADPVGRLVLGIFGCLD------DERARLSDATCTALQVANH--DIDRDLALGRIYVPRA

3vj8A/1-293 VAGLVGIGLSRLFSASEFEDPLVGEDTERANSMGLFLQKTNIIRDYLEDQQGGREFWPQE

2nd_struc_prediction/1-349 HHHHHHHHHHHHHCCCCCCCCCHHHHHHHHHHHHHHHHHHHHHHHHHHHHHCCCSSCCHH

PSY_A/1-349 ELAQAGLSDEDIFAGKVTDKWRNFMKMQLKRARMFFDEAEKGITELDAASRWPVWASLLL

2zcsA/1-281 RLKQYEVDIAEVYQNGVNNHYIDLWEYYAAIAEKDFRDVMDQIKVFSIEAQPIIELAARI

3acxA/1-277 RLKQYEVDIAEVYQNGVNNHYIDLWEYYAAIAEKDFRDVMDQIKVFSIEAQPIIELAARI

4e9uA/1-281 RLKQYEVDIAEVYQNGVNNHYIDLWEYYAAIAEKDFRDVMDQIKVFSIEAQPIIELAARI

2zcoA/1-281 RLKQYEVDIAEVYQNGVNNHYIDLWEYYAAIAEKDFRDVMDQIKVFSIEAQPIIELAARI

2zcsA/1-281 RLKQYEVDIAEVYQNGVNNHYIDLWEYYAAIAEKDFRDVMDQIKVFSIEAQPIIELAARI

3acxA/1-277 RLKQYEVDIAEVYQNGVNNHYIDLWEYYAAIAEKDFRDVMDQIKVFSIEAQPIIELAARI

4r9uA/1-281 RLKQYEVDIAEVYQNGVNNHYIDLWEYYAAIAEKDFRDVMDQIKVFSIEAQPIIELAARI

2zcsA/1-275 RLKQYEVDIAEVYQNGVNNHYIDLWEYYAAIAEKDFRDVMDQIKVFSIEAQPIIELAARI

4hdlA/1-258 DLEQFGATLDDIRARRATDGVRRCIALEVDRAQALFDEGRRLESLVPPRLARQLKLYRLG

3vj8A/1-293 VWSRYVKKLGDFAKPENIDLAVQCLNELITNALHHIPDVITYLSRLRNQSVFNFCIPQVM

2nd_struc_prediction/1-349 HHHHCCCCHHHHHCCCCCHHHHHHHHHHHHHHHHHHHHHHHHHHHCCHHHHHHHHHHHHH

PSY_A/1-349 YRRILDEIEANDYNNFTKRAYVGKAKKIAALPLAYAKSVLKTPSSRGTT

2zcsA/1-281 YIEILDEVRQANY-TLHERVFVEKRKKAKLFHEINSKY-----------

3acxA/1-277 YIEILDEVRQANYT-LHERVFVEKRKKAKLFHEINSKY-----------

4e9uA/1-281 YIEILDEVRQANYT-LHERVFVEKRKKAKLFHEINSKY-----------

2zcoA/1-281 YIEILDEVRQANYT-LHERVFVEKRKKAKLFHEIN-----------SKY

2zcsA/1-281 YIEILDEVRQANYT-LHERVFVEKRKKAKLFHEINSKY-----------

3acxA/1-277 YIEILDEVRQANYT-LHERVFVEKRKKAKLFHEINSKY-----------

4r9uA/1-281 YIEILDEVRQANY-TLHERVFVEKRKKAKLFHEINSKY-----------

2zcsA/1-275 YIEILDEVRQANY-TLHERVFVEKRKKAKLFHEINSKY-----------

4hdlA/1-258 GEAILAAIRRQGYNPF---------------------------------

3vj8A/1-293 AIATLAACY-NNQQVFKGAVKIRKGQAVTLMMDATNMPIYHRIPDSDP-

2nd_struc_prediction/1-349 HHHHHHHHHHCCCCCCCCCCCCCHHHHHHHHHHHHHHHHCCCCCCCCCC

**BnaA.PSY.b**

PSY_B/1-349 ASHAGEIALSSEEKVYNVVLRQAALVNKQLRSTSPELDDVKKPQDIVLPGSLSLLGEAYD

2zcsA/1-281 ---------------------------------------------------MTMMDMNFK

3acxA/1-277 ---------------------------------------------------MTMMDMNFK

4e9uA/1-281 ---------------------------------------------------MTMMDMNFK

2zcoA/1-281 MT---------------------------------------------------MMDMNFK

2zcsA/1-281 ---------------------------------------------------MTMMDMNFK

3acxA/1-277 ---------------------------------------------------MTMMDMNFK

4e9uA/1-281 ---------------------------------------------------MTMMDMNFK

2zcsA/1-270 ---------------------------------------------------------NFK

4hd1A/1-258 ----------------------------------------------NAGSVPVELRGDFE

3vj8A/1-293 -----------------------------------------------------SLSSSLK

2nd_struct_predic/1-349 CCCCCCCCCCCCCCCCHHHHHHHHHHHHCCCCCCCCCCCCCCCCCCCCCCCCCCHHHHHH

PSY_B/1-349 RCGEVCAEYAKTFYLGTLLMTPERRKAIWAIYVWCRRTDELVDGPNASHITPMALDRWEA

2zcsA/1-281 YCHKIMKKHSKSFSYAFDLLPEDQRKAVWAIYAVCRKIDDSIDVYGDINQIKEDIQSIEK

3acxA/1-277 YCHKIMKKHSKSFSYAFDLLPEDQRKAVWAIYAVCRKIDDSIDVYGDIQF----LNQIKE

4e9uA/1-281 YCHKIMKKHSKSFSYAFDLLPEDQRKAVWAIYAVCRKIDDSIDVYGDINQIKEDIQSIEK

2zcoA/1-281 YCHKIMKKHSKSFSYAFDLLPEDQRKAVWAIYAVCRKIDDSIDVYGDIQQIKEDIQSIEK

2zcsA/1-281 YCHKIMKKHSKSFSYAFDLLPEDQRKAVWAIYAVCRKIDDSIDVYGDINQIKEDIQSIEK

3acxA/1-277 YCHKIMKKHSKSFSYAFDLLPEDQRKAVWAIYAVCRKIDDSIDVYGDIQF----LNQIKE

4e9uA/1-281 YCHKIMKKHSKSFSYAFDLLPEDQRKAVWAIYAVCRKIDDSIDVYGDINQIKEDIQSIEK

2zcsA/1-270 YCHKIMKKHSKSFSYAFDLLPEDQRKAVWAIYAVCRKIDDSIDVYGDI----QFLNQIKE

4hd1A/1-258 VCRRLTRSHYENFSVVSLFVPRHLRPHFYSVYAFCRGVDDLGDEFAGD---RAALDAYEE

3vj8A/1-293 TCYKYLNQTSRSFAAVIQALDGEMRNAVCIFYLVLRALDTLEDDMTSVEKKVPLLHNFHS

2nd_struct_predic/1-349 HHHHHHHHCCCCHHHHHHHCCHHHHHHHHHHHHHHHHHHHHHCCCCCCHHHHHHHHHHHH

PSY_B/1-349 RLEDLFRGRPFDMLDAALADTVARYPVDIQPFRDMIEGMRMDLRKSRYKNFDDLYLYCYY

2zcsA/1-281 YPYEYHHFQSDRRIMMALQHVAQHKNIAFQSFYNLIDTVYKDQHFTMFETDAELFGYCYG

3acxA/1-277 DIQSIEKYPSDRRIMMALQHVAQHKNIAFQSFYNLIDTVYKDQHFTMFETDAELFGYCYG

4e9uA/1-281 YPYEYHHFQSDRRIMMALQHVAQHKNIAFQSFYNLIDTVYKDQHFTMFETDAELFGYCYG

2zcoA/1-281 YPYEYHHFQSDRRIMMALQHVAQHKNIAFQSFYNLIDTVYKDQHFTMFETDAELFGYCYG

2zcsA/1-281 YPYEYHHFQSDRRIMMALQHVAQHKNIAFQSFYNLIDTVYKDQHFTMFETDAELFGYCYG

3acxA/1-277 DIQSIEKYPSDRRIMMALQHVAQHKNIAFQSFYNLIDTVYKDQHFTMFETDAELFGYCYG

4e9uA/1-281 YPYEYHHFQSDRRIMMALQHVAQHKNIAFQSFYNLIDTVYKDQHFTMFETDAELFGYCYG

2zcsA/1-270 DIQSIEKYQSDRRIMMALQHVAQHKNIAFQSFYNLIDTVYKDQHFTMFETDAELFGYCYG

4hd1A/1-258 ELRRAFAGEATTPAFRALQFTIATCNLP-EPFLRLIEANRRDQRKHTYDTWEDLRDYCRY

3vj8A/1-293 FLYQRFMESKDRQVLEDFPTISLEFRNLAEKYQTVIIGMAEFLD-KHVTSEQEWDKYCHY

2nd_struct_predic/1-349 HHHHHHCCCCCCHHHHHHHHHHHHHCCCHHHHHHHHHHHHHHCCCCCCCCHHHHHHHHHH

PSY_B/1-349 VAGTVGLMSVPVMGIDPKSKATTESVYNAALALGIANQLTNILRDVGEDARRGRVYLPQD

2zcsA/1-281 VAGTVGEVLTPILSDHET-----HQTYDVARRLGESLQLINILRDVGEDFENERIYFSKQ

3acxA/1-277 VAGTVGEVLTPILSDHE-----THQTYDVARRLGESLQLINILRDVGEDFENERIYFSKQ

4e9uA/1-281 VAGTVGEVLTPILSDHET-----HQTYDVARRLGESLQLINILRDVGEDFENERIYFSKQ

2zcoA/1-281 VAGTVGEVLTPILSDHET-----HQTYDVARRLGESLQLINILRDVGEDFENERIYFSKQ

2zcsA/1-281 VAGTVGEVLTPILSDHET-----HQTYDVARRLGESLQLINILRDVGEDFENERIYFSKQ

3acxA/1-277 VAGTVGEVLTPILSDHE-----THQTYDVARRLGESLQLINILRDVGEDFENERIYFSKQ

4e9uA/1-281 VAGTVGEVLTPILSDHET-----HQTYDVARRLGESLQLINILRDVGEDFENERIYFSKQ

2zcsA/1-270 VAGTVGEVLTPILSDHET-----HQTYDVARRLGESLQLINILRDVGEDFENERIYFSKQ

4hd1A/1-258 SADPVGRLVLGIFGCLD------DERARLSDATCTALQVANH--DIDRDLALGRIYVPRA

3vj8A/1-293 VAGLVGIGLSRLFSASEFEDPLVGEDTERANSMGLFLQKTNIIRDYLEDQQGGREFWPQE

2nd_struct_predic/1-349 HHHHHHHHHHHHHCCCCCCCCCHHHHHHHHHHHHHHHHHHHHHHHHHHHHHCCCSSCCHH

PSY_B/1-349 ELAQAGLSDEDIFAGKVTDKWRNFMKMQLKRARMFFDEAEKGVTELDAASRWPVWASLLL

2zcsA/1-281 RLKQYEVDIAEVYQNGVNNHYIDLWEYYAAIAEKDFRDVMDQIKVFSIEAQPIIELAARI

3acxA/1-277 RLKQYEVDIAEVYQNGVNNHYIDLWEYYAAIAEKDFRDVMDQIKVFSIEAQPIIELAARI

4e9uA/1-281 RLKQYEVDIAEVYQNGVNNHYIDLWEYYAAIAEKDFRDVMDQIKVFSIEAQPIIELAARI

2zcoA/1-281 RLKQYEVDIAEVYQNGVNNHYIDLWEYYAAIAEKDFRDVMDQIKVFSIEAQPIIELAARI

2zcsA/1-281 RLKQYEVDIAEVYQNGVNNHYIDLWEYYAAIAEKDFRDVMDQIKVFSIEAQPIIELAARI

3acxA/1-277 RLKQYEVDIAEVYQNGVNNHYIDLWEYYAAIAEKDFRDVMDQIKVFSIEAQPIIELAARI

4e9uA/1-281 RLKQYEVDIAEVYQNGVNNHYIDLWEYYAAIAEKDFRDVMDQIKVFSIEAQPIIELAARI

2zcsA/1-270 RLKQYEVDIAEVYQNGVNNHYIDLWEYYAAIAEKDFRDVMDQIKVFSIEAQPIIELAARI

4hd1A/1-258 DLEQFGATLDDIRARRATDGVRRCIALEVDRAQALFDEGRRLESLVPPRLARQLKLYRLG

3vj8A/1-293 VWSRYVKKLGDFAKPENIDLAVQCLNELITNALHHIPDVITYLSRLRNQSVFNFCIPQVM

2nd_struct_predic/1-349 HHHHCCCCHHHHHCCCCCHHHHHHHHHHHHHHHHHHHHHHHHHHHCCHHHHHHHHHHHHH

PSY_B/1-349 YRRILDEIEANDYNNFTKRAYVGKAKKIAALPLAYAKSVLKTPSSRGTT

2zcsA/1-281 YIEILDEVRQANY-TLHERVFVEKRKKAKLFHEINSKY-----------

3acxA/1-277 YIEILDEVRQANYT-LHERVFVEKRKKAKLFHEINSKY-----------

4e9uA/1-281 YIEILDEVRQANYT-LHERVFVEKRKKAKLFHEINSKY-----------

2zcoA/1-281 YIEILDEVRQANYT-LHERVFVEKRKKAKLFHEIN-----------SKY

2zcsA/1-281 YIEILDEVRQANYT-LHERVFVEKRKKAKLFHEINSKY-----------

3acxA/1-277 YIEILDEVRQANYT-LHERVFVEKRKKAKLFHEINSKY-----------

4e9uA/1-281 YIEILDEVRQANY-TLHERVFVEKRKKAKLFHEINSKY-----------

2zcsA/1-270 YIEILDEVRQANY-TLHERVFVEKRKKAKLFHEINSK------------

4hd1A/1-258 GEAILAAIRRQGYNPF---------------------------------

3vj8A/1-293 AIATLAACY-NNQQVFKGAVKIRKGQAVTLMMDATNMPAVEEIYHRIP-

2nd_struct_predic/1-349 HHHHHHHHHHCCCCCCCCCCCCCHHHHHHHHHHHHHHHHCCCCCCCCCC

**BnaA.PSY.c**

PSY_C/1-346 LVVNPAGEMTLSSEEKVYNVVLSQAALVNEQLRSTSPVLDDVKKPQDTVRPGSLSLLGEA

2zcsA/1-281 -----------------------------------------------------MTMMDMN

3acxA/1-277 -----------------------------------------------------MTMMDMN

4e9uA/1-281 -----------------------------------------------------MTMMDMN

2zcoA/1-281 M-----------------------------------------------------TMMDMN

2zcsA/1-281 -----------------------------------------------------MTMMDMN

3acxA/1-277 -----------------------------------------------------MTMMDMN

4e9uA/1-281 -----------------------------------------------------MTMMDMN

2zcsA/1-271 -----------------------------------------------------------N

4hd1A/1-258 ------------------------------------------------NAGSVPVELRGD

3vj8A/1-288 -------------------------------------------------------SLSSS

2nd_struct_pred/1-346 CCCCCCCCCCCCCCCCHHHHHHHHHHHHHHCCCCCCCCCCCCCCCCCCCCCCCCCCHHHH

PSY_C/1-346 YDRCGEVCAEYAKTFYLGTLLMTPERRKAIWAIYVWCRRTDELVDGPNASHITPMALDRW

2zcsA/1-281 FKYCHKIMKKHSKSFSYAFDLLPEDQRKAVWAIYAVCRKIDDSIDVYGDINQIKEDIQSI

3acxA/1-277 FKYCHKIMKKHSKSFSYAFDLLPEDQRKAVWAIYAVCRKIDDSIDVYGDIQF----LNQI

4e9uA/1-281 FKYCHKIMKKHSKSFSYAFDLLPEDQRKAVWAIYAVCRKIDDSIDVYGDINQIKEDIQSI

2zcoA/1-281 FKYCHKIMKKHSKSFSYAFDLLPEDQRKAVWAIYAVCRKIDDSIDVYGDIQQIKEDIQSI

2zcsA/1-281 FKYCHKIMKKHSKSFSYAFDLLPEDQRKAVWAIYAVCRKIDDSIDVYGDINQIKEDIQSI

3acxA/1-277 FKYCHKIMKKHSKSFSYAFDLLPEDQRKAVWAIYAVCRKIDDSIDVYGDIQF----LNQI

4e9uA/1-281 FKYCHKIMKKHSKSFSYAFDLLPEDQRKAVWAIYAVCRKIDDSIDVYGDINQIKEDIQSI

2zcsA/1-271 FKYCHKIMKKHSKSFSYAFDLLPEDQRKAVWAIYAVCRKIDDSIDVYGDI----QFLNQI

4hd1A/1-258 FEVCRRLTRSHYENFSVVSLFVPRHLRPHFYSVYAFCRGVDDLGDEFAGD---RAALDAY

3vj8A/1-288 LKTCYKYLNQTSRSFAAVIQALDGEMRNAVCIFYLVLRALDTLEDDMTSVEKKVPLLHNF

2nd_struct_pred/1-346 HHHHHHHHHHCCCCHHHHHHHCCHHHHHHHHHHHHHHHHHHHHHCCCCCCHHHHHHHHHH

PSY_C/1-346 EARLEDLYRGRAFDMLDAALADTVTRYPVDIQPFRDMIEGMRMDLRKSRYNNFDELYLYC

2zcsA/1-281 EKYPYEYHHFQSDRRIMMALQHVAQHKNIAFQSFYNLIDTVYKDQHFTMFETDAELFGYC

3acxA/1-277 KEDIQSIEKYPSDRRIMMALQHVAQHKNIAFQSFYNLIDTVYKDQHFTMFETDAELFGYC

4e9uA/1-281 EKYPYEYHHFQSDRRIMMALQHVAQHKNIAFQSFYNLIDTVYKDQHFTMFETDAELFGYC

2zcoA/1-281 EKYPYEYHHFQSDRRIMMALQHVAQHKNIAFQSFYNLIDTVYKDQHFTMFETDAELFGYC

2zcsA/1-281 EKYPYEYHHFQSDRRIMMALQHVAQHKNIAFQSFYNLIDTVYKDQHFTMFETDAELFGYC

3acxA/1-277 KEDIQSIEKYPSDRRIMMALQHVAQHKNIAFQSFYNLIDTVYKDQHFTMFETDAELFGYC

4e9uA/1-281 EKYPYEYHHFQSDRRIMMALQHVAQHKNIAFQSFYNLIDTVYKDQHFTMFETDAELFGYC

2zcsA/1-271 KEDIQSIEKFQSDRRIMMALQHVAQHKNIAFQSFYNLIDTVYKDQHFTMFETDAELFGYC

4hd1A/1-258 EEELRRAFAGEATTPAFRALQFTIATCNLP-EPFLRLIEANRRDQRKHTYDTWEDLRDYC

3vj8A/1-288 HSFLYQRFMESKDRQVLEDFPTISLEFRNLAEKYQTVIIGMAEFLD-KHVTSEQEWDKYC

2nd_struct_pred/1-346 HHHHHHHHCCCCCCHHHHHHHHHHHHHCCCHHHHHHHHHHHHHHHCCCCCCCHHHHHHHH

PSY_C/1-346 YYVAGTVGLMSVPVMGIDPKSKATTESVYNAALALGIANQLTNILRDVGEDARRGRVYLP

2zcsA/1-281 YGVAGTVGEVLTPILSDHET-----HQTYDVARRLGESLQLINILRDVGEDFENERIYFS

3acxA/1-277 YGVAGTVGEVLTPILSDHET-----HQTYDVARRLGESLQLINILRDVGEDFENERIYFS

4e9uA/1-281 YGVAGTVGEVLTPILSDHET-----HQTYDVARRLGESLQLINILRDVGEDFENERIYFS

2zcoA/1-281 YGVAGTVGEVLTPILSDHET-----HQTYDVARRLGESLQLINILRDVGEDFENERIYFS

2zcsA/1-281 YGVAGTVGEVLTPILSDHET-----HQTYDVARRLGESLQLINILRDVGEDFENERIYFS

3acxA/1-277 YGVAGTVGEVLTPILSDHE-----THQTYDVARRLGESLQLINILRDVGEDFENERIYFS

4e9uA/1-281 YGVAGTVGEVLTPILSDHET-----HQTYDVARRLGESLQLINILRDVGEDFENERIYFS

2zcsA/1-271 YGVAGTVGEVLTPILSDHET-----HQTYDVARRLGESLQLINILRDVGEDFENERIYFS

4hd1A/1-258 RYSADPVGRLVLGIFGCLD------DERARLSDATCTALQVANH--DIDRDLALGRIYVP

3vj8A/1-288 HYVAGLVGIGLSRLFSASEFEDPLVGEDTERANSMGLFLQKTNIIRDYLEDQQGGREFWP

2nd_struct_pred/1-346 HHHHHHHHHHHHHHHCCCCCCCCCHHHHHHHHHHHHHHHHHHHHHHHCHHHHHCCCSSCC

PSY_C/1-346 QDELAQAGLSDEDIFAGKVTDKWRNFMRMQLKRARMFFDEAEKGVTELDAASRWPVWASL

2zcsA/1-281 KQRLKQYEVDIAEVYQNGVNNHYIDLWEYYAAIAEKDFRDVMDQIKVFSIEAQPIIELAA

3acxA/1-277 KQRLKQYEVDIAEVYQNGVNNHYIDLWEYYAAIAEKDFRDVMDQIKVFSIEAQPIIELAA

4e9uA/1-281 KQRLKQYEVDIAEVYQNGVNNHYIDLWEYYAAIAEKDFRDVMDQIKVFSIEAQPIIELAA

2zcoA/1-281 KQRLKQYEVDIAEVYQNGVNNHYIDLWEYYAAIAEKDFRDVMDQIKVFSIEAQPIIELAA

2zcsA/1-281 KQRLKQYEVDIAEVYQNGVNNHYIDLWEYYAAIAEKDFRDVMDQIKVFSIEAQPIIELAA

3acxA/1-277 KQRLKQYEVDIAEVYQNGVNNHYIDLWEYYAAIAEKDFRDVMDQIKVFSIEAQPIIELAA

4e9uA/1-281 KQRLKQYEVDIAEVYQNGVNNHYIDLWEYYAAIAEKDFRDVMDQIKVFSIEAQPIIELAA

2zcsA/1-271 KQRLKQYEVDIAEVYQNGVNNHYIDLWEYYAAIAEKDFRDVMDQIKVFSIEAQPIIELAA

4hd1A/1-258 RADLEQFGATLDDIRARRATDGVRRCIALEVDRAQALFDEGRRLESLVPPRLARQLKLYR

3vj8A/1-288 QEVWSRYVKKLGDFAKPENIDLAVQCLNELITNALHHIPDVITYLSRLRNQSVFNFCIPQ

2nd_struct_pred/1-346 HHHHHHCCCCHHHHHCCCCCHHHHHHHHHHHHHHHHHHHHHHHHHHHCCHHHHHHHHHHH

PSY_C/1-346 LLYRRILDEIEANDYNNFTKRAYVGKVKKIAALPLAYAKSVLKVSS

2zcsA/1-281 RIYIEILDEVRQANY-TLHERVFVEKRKKAKLFHEINSKY------

3acxA/1-277 RIYIEILDEVRQANYT-LHERVFVEKRKKAKLFHEINSKY------

4e9uA/1-281 RIYIEILDEVRQANYT-LHERVFVEKRKKAKLFHEINSKY------

2zcoA/1-281 RIYIEILDEVRQANYT-LHERVFVEKRKKAKLFHEINSK------Y

2zcsA/1-281 RIYIEILDEVRQANYT-LHERVFVEKRKKAKLFHEINSKY------

3acxA/1-277 RIYIEILDEVRQANYT-LHERVFVEKRKKAKLFHEINSKY------

4e9uA/1-281 RIYIEILDEVRQANY-TLHERVFVEKRKKAKLFHEINSKY------

2zcsA/1-271 RIYIEILDEVRQANY-TLHERVFVEKRKKAKLFHEINSKY------

4hd1A/1-258 LGGEAILAAIRRQGYNPF----------------------------

3vj8A/1-288 VMAIATLAACY-NNQQVFKGAVKIRKGQAVTLMMDATNMPAVKDS-

2nd_struct_pred/1-346 HHHHHHHHHHHHCCCCCCCCCCCCCHHHHHHHHHHHHHHHHHCCCC

**BnaA.PSY.d**

PSY_D/1-351 SSLVASPAAGEITLSSEEKVYNVVLKQAALGNKQLRDLDDVKKPQDIVLPGTTTGSLSLL

2zcsA/1-281 --------------------------------------------------------MTMM

3acxA/1-277 --------------------------------------------------------MTMM

4e9uA/1-281 --------------------------------------------------------MTMM

2zcoA/1-281 --------------------------------------------------------MTMM

2zcsA/1-281 --------------------------------------------------------MTMM

3acxA/1-276 --------------------------------------------------------MTMM

4e9uA/1-281 --------------------------------------------------------MTMM

2zcsA/1-271 ------------------------------------------------------------

4hd1A/1-258 ---------------------------------------------------NAGSVPVEL

3vj8A/1-290 ----------------------------------------------------------SL

2nd_struct_pred/1-351 CCCCCCCCCCCCCCCCCCCHCHHHHHHHHHHHHHHCCCCCCCCCCCCCCCCCCCCCCCCC

PSY_D/1-351 GEAYDRCGEVCAEYAKTFYLGTLLMTPERRKAIWAIYVWCRRTDELVDGPNASHITPMAL

2zcsA/1-281 DMNFKYCHKIMKKHSKSFSYAFDLLPEDQRKAVWAIYAVCRKIDDSIDVYGDINQIKEDI

3acxA/1-277 DMNFKYCHKIMKKHSKSFSYAFDLLPEDQRKAVWAIYAVCRKIDDSIDVYGDIQF----L

4e9uA/1-281 DMNFKYCHKIMKKHSKSFSYAFDLLPEDQRKAVWAIYAVCRKIDDSIDVYGDINQIKEDI

2zcoA/1-281 DMNFKYCHKIMKKHSKSFSYAFDLLPEDQRKAVWAIYAVCRKIDDSIDVYGDIQQIKEDI

2zcsA/1-281 DMNFKYCHKIMKKHSKSFSYAFDLLPEDQRKAVWAIYAVCRKIDDSIDVYGDINQIKEDI

3acxA/1-276 DMNFKYCHKIMKKHSKSFSYAFDLLPEDQRKAVWAIYAVCRKIDDSIDVYGDIQF----L

4e9uA/1-281 DMNFKYCHKIMKKHSKSFSYAFDLLPEDQRKAVWAIYAVCRKIDDSIDVYGDINQIKEDI

2zcsA/1-271 --NFKYCHKIMKKHSKSFSYAFDLLPEDQRKAVWAIYAVCRKIDDSIDVYGDI----QFL

4hd1A/1-258 RGDFEVCRRLTRSHYENFSVVSLFVPRHLRPHFYSVYAFCRGVDDLGDEFAGD---RAAL

3vj8A/1-290 SSSLKTCYKYLNQTSRSFAAVIQALDGEMRNAVCIFYLVLRALDTLEDDMTSVEKKVPLL

2nd_struct_pred/1-351 HHHHHHHHHHHHCCCCCHHHHHHHCCHHHHHHHHHHHHHHHHHCHHHCCCCCCHHHHHHH

PSY_D/1-351 DRWEARLEDLFRGRPFDMLDAALADTVARYPVDIQPFRDMVEGMRMDLRKSRYKNFDDLY

2zcsA/1-281 QSIEKYPYEYHHFQSDRRIMMALQHVAQHKNIAFQSFYNLIDTVYKDQHFTMFETDAELF

3acxA/1-277 NQIKEDIQSIEKYPSDRRIMMALQHVAQHKNIAFQSFYNLIDTVYKDQHFTMFETDAELF

4e9uA/1-281 QSIEKYPYEYHHFQSDRRIMMALQHVAQHKNIAFQSFYNLIDTVYKDQHFTMFETDAELF

2zcoA/1-281 QSIEKYPYEYHHFQSDRRIMMALQHVAQHKNIAFQSFYNLIDTVYKDQHFTMFETDAELF

2zcsA/1-281 QSIEKYPYEYHHFQSDRRIMMALQHVAQHKNIAFQSFYNLIDTVYKDQHFTMFETDAELF

3acxA/1-276 NQIKEDIQSIEKYPSDRRIMMALQHVAQHKNIAFQSFYNLIDTVYKDQHFTMFETDAELF

4e9uA/1-281 QSIEKYPYEYHHFQSDRRIMMALQHVAQHKNIAFQSFYNLIDTVYKDQHFTMFETDAELF

2zcsA/1-271 NQIKEDIQSIEKYPYYHHIMMALQHVAQHKNIAFQSFYNLIDTVYKDQHFTMFETDAELF

4hd1A/1-258 DAYEEELRRAFAGEATTPAFRALQFTIATCNLP-EPFLRLIEANRRDQRKHTYDTWEDLR

3vj8A/1-290 HNFHSFLYQRFMESKDRQVLEDFPTISLEFRNLAEKYQTVIIGMAEFLD-KHVTSEQEWD

2nd_struct_pred/1-351 HHHHHHHHHHHCCCCCCHHHHHHHHHHHHHCCCHHHHHHHHHHHHHHCCCCCCCCHHHHH

PSY_D/1-351 LYCYYVAGTVGLMSVPVMGIDPKSKATTESVYNAALALGIANQLTNILRDVGEDARRGRV

2zcsA/1-281 GYCYGVAGTVGEVLTPILSDHET-----HQTYDVARRLGESLQLINILRDVGEDFENERI

3acxA/1-277 GYCYGVAGTVGEVLTPILSDHE-----THQTYDVARRLGESLQLINILRDVGEDFENERI

4e9uA/1-281 GYCYGVAGTVGEVLTPILSDHET-----HQTYDVARRLGESLQLINILRDVGEDFENERI

2zcoA/1-281 GYCYGVAGTVGEVLTPILSDHET-----HQTYDVARRLGESLQLINILRDVGEDFENERI

2zcsA/1-281 GYCYGVAGTVGEVLTPILSDHET-----HQTYDVARRLGESLQLINILRDVGEDFENERI

3acxA/1-276 GYCYGVAGTVGEVLTPILSD------HEHQTYDVARRLGESLQLINILRDVGEDFENERI

4e9uA/1-281 GYCYGVAGTVGEVLTPILSDHET-----HQTYDVARRLGESLQLINILRDVGEDFENERI

2zcsA/1-271 GYCYGVAGTVGEVLTPILSDHET-----HQTYDVARRLGESLQLINILRDVGEDFENERI

4hd1A/1-258 DYCRYSADPVGRLVLGIFGCLD------DERARLSDATCTALQVANH--DIDRDLALGRI

3vj8A/1-290 KYCHYVAGLVGIGLSRLFSASEFEDPLVGEDTERANSMGLFLQKTNIIRDYLEDQQGGRE

2nd_struct_pred/1-351 HHHHHHHHHHHHHHHHHHCCCCCCCCCHHHHHHHHHHHHHHHHHHHHHHHHHHHHHCCCS

PSY_D/1-351 YLPQDELAQAGLSDEDIFAGKVTDKWRNFMKMQLKRARMFFDEAEKGVTELDAASRWPVW

2zcsA/1-281 YFSKQRLKQYEVDIAEVYQNGVNNHYIDLWEYYAAIAEKDFRDVMDQIKVFSIEAQPIIE

3acxA/1-277 YFSKQRLKQYEVDIAEVYQNGVNNHYIDLWEYYAAIAEKDFRDVMDQIKVFSIEAQPIIE

4e9uA/1-281 YFSKQRLKQYEVDIAEVYQNGVNNHYIDLWEYYAAIAEKDFRDVMDQIKVFSIEAQPIIE

2zcoA/1-281 YFSKQRLKQYEVDIAEVYQNGVNNHYIDLWEYYAAIAEKDFRDVMDQIKVFSIEAQPIIE

2zcsA/1-281 YFSKQRLKQYEVDIAEVYQNGVNNHYIDLWEYYAAIAEKDFRDVMDQIKVFSIEAQPIIE

3acxA/1-276 YFSKQRLKQYEVDIAEVYQNGVNNHYIDLWEYYAAIAEKDFRDVMDQIKVFSIEAQPIIE

4e9uA/1-281 YFSKQRLKQYEVDIAEVYQNGVNNHYIDLWEYYAAIAEKDFRDVMDQIKVFSIEAQPIIE

2zcsA/1-271 YFSKQRLKQYEVDIAEVYQNGVNNHYIDLWEYYAAIAEKDFRDVMDQIKVFSIEAQPIIE

4hd1A/1-258 YVPRADLEQFGATLDDIRARRATDGVRRCIALEVDRAQALFDEGRRLESLVPPRLARQLK

3vj8A/1-290 FWPQEVWSRYVKKLGDFAKPENIDLAVQCLNELITNALHHIPDVITYLSRLRNQSVFNFC

2nd_struct_pred/1-351 SCCHHHHHHCCCCHHHHHCCCCCHHHHHHHHHHHHHHHHHHHHHHHHHHHCCHHHHHHHH

PSY_D/1-351 ASLLLYRRILDEIEANDYNNFTKRAYVGKAKKIAALPLAYAKSILKASSSR

2zcsA/1-281 LAARIYIEILDEVRQANY-TLHERVFVEKRKKAKLFHEINSKY--------

3acxA/1-277 LAARIYIEILDEVRQANYT-LHERVFVEKRKKAKLFHEINSKY--------

4e9uA/1-281 LAARIYIEILDEVRQANYT-LHERVFVEKRKKAKLFHEINSKY--------

2zcoA/1-281 LAARIYIEILDEVRQANYT-LHERVFVEKRKKAKLFHEIN--------SKY

2zcsA/1-281 LAARIYIEILDEVRQANYT-LHERVFVEKRKKAKLFHEINSKY--------

3acxA/1-276 LAARIYIEILDEVRQANYT-LHERVFVEKRKKAKLFHEINSKY--------

4e9uA/1-281 LAARIYIEILDEVRQANY-TLHERVFVEKRKKAKLFHEINSKY--------

2zcsA/1-271 LAARIYIEILDEVRQANY-TLHERVFVEKRKKAKLFHEINSKY--------

4hd1A/1-258 LYRLGGEAILAAIRRQGYNPF------------------------------

3vj8A/1-290 AIQVMAIATLAACY-NNQQVFKGAVKIRKGQAVTLMMDATNMPAVKAIPD-

2nd_struct_pred/1-351 HHHHHHHHHHHHHHHCCCCCCCCCCCCCHHHHHHHHHHHHHHHHHHHHCCC

**BnaC.PSY.e**

PSY_E/1-349 SSLLVAHPAGEIDLSSEEKVYNVVLRQAALVNKQLRSTSPVLDDVKKPQDIVRPGSLSLL

2zcsA/1-281 --------------------------------------------------------MTMM

3acxA/1-277 --------------------------------------------------------MTMM

4e9uA/1-281 --------------------------------------------------------MTMM

2zcoA/1-281 MT--------------------------------------------------------MM

2zcsA/1-281 --------------------------------------------------------MTMM

3acxA/1-277 --------------------------------------------------------MTMM

4e9uA/1-281 --------------------------------------------------------MTMM

2zcsA/1-275 ------------------------------------------------------------

4hd1A/1-258 ---------------------------------------------------NAGSVPVEL

3vj8A/1-288 ----------------------------------------------------------SL

2nd_struct_pred/1-349 CCCCCSCCCCCCCCCCCCCHHHHHHHHHHHHHHHCCCCCCCCCHCCCCCCCCCCCCCCCH

PSY_E/1-349 AEAYDRCGEVCAEYAKTFYLGTLLMTPERRKAIWAIYVWCRRTDELVDGPNASHITPMAL

2zcsA/1-281 DMNFKYCHKIMKKHSKSFSYAFDLLPEDQRKAVWAIYAVCRKIDDSIDVYGDINQIKEDI

3acxA/1-277 DMNFKYCHKIMKKHSKSFSYAFDLLPEDQRKAVWAIYAVCRKIDDSIDVYGDIQF----L

4e9uA/1-281 DMNFKYCHKIMKKHSKSFSYAFDLLPEDQRKAVWAIYAVCRKIDDSIDVYGDINQIKEDI

2zcoA/1-281 DMNFKYCHKIMKKHSKSFSYAFDLLPEDQRKAVWAIYAVCRKIDDSIDVYGDINQIKEDI

2zcsA/1-281 DMNFKYCHKIMKKHSKSFSYAFDLLPEDQRKAVWAIYAVCRKIDDSIDVYGDINQIKEDI

3acxA/1-277 DMNFKYCHKIMKKHSKSFSYAFDLLPEDQRKAVWAIYAVCRKIDDSIDVYGDIQF----L

4e9uA/1-281 DMNFKYCHKIMKKHSKSFSYAFDLLPEDQRKAVWAIYAVCRKIDDSIDVYGDINQIKEDI

2zcsA/1-275 --NFKYCHKIMKKHSKSFSYAFDLLPEDQRKAVWAIYAVCRKIDDSIDVYGDIQFLNQIK

4hd1A/1-258 RGDFEVCRRLTRSHYENFSVVSLFVPRHLRPHFYSVYAFCRGVDDLGDEFAGD---RAAL

3vj8A/1-288 SSSLKTCYKYLNQTSRSFAAVIQALDGEMRNAVCIFYLVLRALDTLEDDMTSVEKKVPLL

2nd_struct_pred/1-349 HHHHHHHHHHHHCCCCCHHHHHHHCCHHHHHHHHHHHHHHHHHCHHCCCCCCCHHHHHHH

PSY_E/1-349 DRWEARLEDLFHGRAFDMLDAALADTVTRYPVDIQPFRDMIEGMRMDLRKSRYNNFDELY

2zcsA/1-281 QSIEKYPYEYHHFQSDRRIMMALQHVAQHKNIAFQSFYNLIDTVYKDQHFTMFETDAELF

3acxA/1-277 NQIKEDIQSIEKYPSDRRIMMALQHVAQHKNIAFQSFYNLIDTVYKDQHFTMFETDAELF

4e9uA/1-281 QSIEKYPYEYHHFQSDRRIMMALQHVAQHKNIAFQSFYNLIDTVYKDQHFTMFETDAELF

2zcoA/1-281 QSIEKYPYEYHHFQSDRRIMMALQHVAQHKNIAFQSFYNLIDTVYKDQHFTMFETDAELF

2zcsA/1-281 QSIEKYPYEYHHFQSDRRIMMALQHVAQHKNIAFQSFYNLIDTVYKDQHFTMFETDAELF

3acxA/1-277 NQIKEDIQSIEKYPSDRRIMMALQHVAQHKNIAFQSFYNLIDTVYKDQHFTMFETDAELF

4e9uA/1-281 QSIEKYPYEYHHFQSDRRIMMALQHVAQHKNIAFQSFYNLIDTVYKDQHFTMFETDAELF

2zcsA/1-275 EDIQSIEKYPYEYHSDRRIMMALQHVAQHKNIAFQSFYNLIDTVYKDQHFTMFETDAELF

4hd1A/1-258 DAYEEELRRAFAGEATTPAFRALQFTIATCNLP-EPFLRLIEANRRDQRKHTYDTWEDLR

3vj8A/1-288 HNFHSFLYQRFMESKDRQVLEDFPTISLEFRNLAEKYQTVIIGMAEFLD-KHVTSEQEWD

2nd_struct_pred/1-349 HHHHHHHHHHHCCCCCCHHHHHHHHHHHHHCCCHHHHHHHHHHHHHHHCCCCCCCHHHHH

PSY_E/1-349 LYCYYVAGTVGLMSVPVMGINPKSKATTESVYNAALALGIANQLTNILRDVGEDARRGRV

2zcsA/1-281 GYCYGVAGTVGEVLTPILSDHET-----HQTYDVARRLGESLQLINILRDVGEDFENERI

3acxA/1-277 GYCYGVAGTVGEVLTPILSDHET-----HQTYDVARRLGESLQLINILRDVGEDFENERI

4e9uA/1-281 GYCYGVAGTVGEVLTPILSDHET-----HQTYDVARRLGESLQLINILRDVGEDFENERI

2zcoA/1-281 GYCYGVAGTVGEVLTPILSDHET-----HQTYDVARRLGESLQLINILRDVGEDFENERI

2zcsA/1-281 GYCYGVAGTVGEVLTPILSDHET-----HQTYDVARRLGESLQLINILRDVGEDFENERI

3acxA/1-277 GYCYGVAGTVGEVLTPILSDHET-----HQTYDVARRLGESLQLINILRDVGEDFENERI

4e9uA/1-281 GYCYGVAGTVGEVLTPILSDHET-----HQTYDVARRLGESLQLINILRDVGEDFENERI

2zcsA/1-275 GYCYGVAGTVGEVLTPILSDHET-----HQTYDVARRLGESLQLINILRDVGEDFENERI

4hd1A/1-258 DYCRYSADPVGRLVLGIFGCLD------DERARLSDATCTALQVANH--DIDRDLALGRI

3vj8A/1-288 KYCHYVAGLVGIGLSRLFSASEFEDPLVGEDTERANSMGLFLQKTNIIRDYLEDQQGGRE

2nd_struct_pred/1-349 HHHHHHHHHHHHHHHHHHCCCCCCCCCHHHHHHHHHHHHHHHHHHHHHHHHHHHHHCCCS

PSY_E/1-349 YLPQDELVQAGLSDEDIFAGKVTDKWRNFMRLQLKRARMFFDEAEKGVTELDAASRWPVW

2zcsA/1-281 YFSKQRLKQYEVDIAEVYQNGVNNHYIDLWEYYAAIAEKDFRDVMDQIKVFSIEAQPIIE

3acxA/1-277 YFSKQRLKQYEVDIAEVYQNGVNNHYIDLWEYYAAIAEKDFRDVMDQIKVFSIEAQPIIE

4e9uA/1-281 YFSKQRLKQYEVDIAEVYQNGVNNHYIDLWEYYAAIAEKDFRDVMDQIKVFSIEAQPIIE

2zcoA/1-281 YFSKQRLKQYEVDIAEVYQNGVNNHYIDLWEYYAAIAEKDFRDVMDQIKVFSIEAQPIIE

2zcsA/1-281 YFSKQRLKQYEVDIAEVYQNGVNNHYIDLWEYYAAIAEKDFRDVMDQIKVFSIEAQPIIE

3acxA/1-277 YFSKQRLKQYEVDIAEVYQNGVNNHYIDLWEYYAAIAEKDFRDVMDQIKVFSIEAQPIIE

4e9uA/1-281 YFSKQRLKQYEVDIAEVYQNGVNNHYIDLWEYYAAIAEKDFRDVMDQIKVFSIEAQPIIE

2zcsA/1-275 YFSKQRLKQYEVDIAEVYQNGVNNHYIDLWEYYAAIAEKDFRDVMDQIKVFSIEAQPIIE

4hd1A/1-258 YVPRADLEQFGATLDDIRARRATDGVRRCIALEVDRAQALFDEGRRLESLVPPRLARQLK

3vj8A/1-288 FWPQEVWSRYVKKLGDFAKPENIDLAVQCLNELITNALHHIPDVITYLSRLRNQSVFNFC

2nd_struct_pred/1-349 SCCHHHHHHCCCCHHHHHCCCCCHHHHHHHHHHHHHHHHHHHHHHHHHHHCCHHHHHHHH

PSY_E/1-349 ASLLLYRRILDEIEANDYNNFTKRAYVGKVKKIAALPLAYAKSVVKVSS

2zcsA/1-281 LAARIYIEILDEVRQANY-TLHERVFVEKRKKAKLFHEINSKY------

3acxA/1-277 LAARIYIEILDEVRQANYT-LHERVFVEKRKKAKLFHEINSKY------

4e9uA/1-281 LAARIYIEILDEVRQANYT-LHERVFVEKRKKAKLFHEINSKY------

2zcoA/1-281 LAARIYIEILDEVRQANYT-LHERVFVEKRKKAKLFHEIN------SKY

2zcsA/1-281 LAARIYIEILDEVRQANYT-LHERVFVEKRKKAKLFHEINSKY------

3acxA/1-277 LAARIYIEILDEVRQANYT-LHERVFVEKRKKAKLFHEINSKY------

4e9uA/1-281 LAARIYIEILDEVRQANY-TLHERVFVEKRKKAKLFHEINSKY------

2zcsA/1-275 LAARIYIEILDEVRQANY-TLHERVFVEKRKKAKLFHEINSKY------

4hd1A/1-258 LYRLGGEAILAAIRRQGYNPF----------------------------

3vj8A/1-288 IPQVMAIATLAACY-NNQQVFKGAVKIRKGQAVTLMMDATNMPAVPDS-

2nd_struct_pred/1-349 HHHHHHHHHHHHHHHCCCCCCCCCCCCCHHHHHHHHHHHHHHHHCCCCC

**BnaC.PSY.f**

PSY_F/1-351 SSLVASPAAGEITLSSEEKVYNVVLKQAALVNKQLRDLDDLKKPQDIVLPGTTTGSLSLL

2zcsA/1-281 --------------------------------------------------------MTMM

3acxA/1-277 --------------------------------------------------------MTMM

4e9uA/1-281 --------------------------------------------------------MTMM

2zcoA/1-281 --------------------------------------------------------MTMM

2zcsA/1-281 --------------------------------------------------------MTMM

3acxA/1-277 --------------------------------------------------------MTMM

4e9uA/1-281 --------------------------------------------------------MTMM

2zcsA/1-270 ------------------------------------------------------------

4hd1A/1-258 ---------------------------------------------------NAGSVPVEL

3vj8A/1-290 ----------------------------------------------------------SL

2nd_struc_pred/1-351 CCCCCCCCCCCCCCCCCCHHHHHHHHHHHHHHHHHCCCCCCCCCCCCCCCCCCCCCCCCH

PSY_F/1-351 GEAYDRCGEVCAEYAKTFYLGTLLMTPERRKAIWAIYVWCRRTDELVDGPNASHITPMAL

2zcsA/1-281 DMNFKYCHKIMKKHSKSFSYAFDLLPEDQRKAVWAIYAVCRKIDDSIDVYGDINQIKEDI

3acxA/1-277 DMNFKYCHKIMKKHSKSFSYAFDLLPEDQRKAVWAIYAVCRKIDDSIDVYGDIQF----L

4e9uA/1-281 DMNFKYCHKIMKKHSKSFSYAFDLLPEDQRKAVWAIYAVCRKIDDSIDVYGDINQIKEDI

2zcoA/1-281 DMNFKYCHKIMKKHSKSFSYAFDLLPEDQRKAVWAIYAVCRKIDDSIDVYGDIQQIKEDI

2zcsA/1-281 DMNFKYCHKIMKKHSKSFSYAFDLLPEDQRKAVWAIYAVCRKIDDSIDVYGDINQIKEDI

3acxA/1-277 DMNFKYCHKIMKKHSKSFSYAFDLLPEDQRKAVWAIYAVCRKIDDSIDVYGDIQF----L

4e9uA/1-281 DMNFKYCHKIMKKHSKSFSYAFDLLPEDQRKAVWAIYAVCRKIDDSIDVYGDINQIKEDI

2zcsA/1-270 --NFKYCHKIMKKHSKSFSYAFDLLPEDQRKAVWAIYAVCRKIDDSIDVYGDI----QFL

4hd1A/1-258 RGDFEVCRRLTRSHYENFSVVSLFVPRHLRPHFYSVYAFCRGVDDLGDEFAGD---RAAL

3vj8A/1-290 SSSLKTCYKYLNQTSRSFAAVIQALDGEMRNAVCIFYLVLRALDTLEDDMTSVEKKVPLL

2nd_struc_pred/1-351 HHHHHHHHHHHHCCCCCHHHHHHHCCHHHHHHHHHHHHHHHHHHHHHCCCCCCHHHHHHH

PSY_F/1-351 DRWEARLEDLFRGRPFDMLDAALADTVARYPVDVQPFRDMVEGMRMDLRKSRYKNFDDLY

2zcsA/1-281 QSIEKYPYEYHHFQSDRRIMMALQHVAQHKNIAFQSFYNLIDTVYKDQHFTMFETDAELF

3acxA/1-277 NQIKEDIQSIEKYPSDRRIMMALQHVAQHKNIAFQSFYNLIDTVYKDQHFTMFETDAELF

4e9uA/1-281 QSIEKYPYEYHHFQSDRRIMMALQHVAQHKNIAFQSFYNLIDTVYKDQHFTMFETDAELF

2zcoA/1-281 QSIEKYPYEYHHFQSDRRIMMALQHVAQHKNIAFQSFYNLIDTVYKDQHFTMFETDAELF

2zcsA/1-281 QSIEKYPYEYHHFQSDRRIMMALQHVAQHKNIAFQSFYNLIDTVYKDQHFTMFETDAELF

3acxA/1-277 NQIKEDIQSIEKYPSDRRIMMALQHVAQHKNIAFQSFYNLIDTVYKDQHFTMFETDAELF

4e9uA/1-281 QSIEKYPYEYHHFQSDRRIMMALQHVAQHKNIAFQSFYNLIDTVYKDQHFTMFETDAELF

2zcsA/1-270 NQIKEDIQSIEKYPYYHHIMMALQHVAQHKNIAFQSFYNLIDTVYKDQHFTMFETDAELF

4hd1A/1-258 DAYEEELRRAFAGEATTPAFRALQFTIATCNLP-EPFLRLIEANRRDQRKHTYDTWEDLR

3vj8A/1-290 HNFHSFLYQRFMESKDRQVLEDFPTISLEFRNLAEKYQTVIIGMAEFLD-KHVTSEQEWD

2nd_struc_pred/1-351 HHHHHHHHHHHCCCCCCHHHHHHHHHHHHHCCCHHHHHHHHHHHHHHCCCCCCCCHHHHH

PSY_F/1-351 LYCYYVAGTVGLMSVPVMGIDPKSKATTESVYNAALALGIANQLTNILRDVGEDARRGRV

2zcsA/1-281 GYCYGVAGTVGEVLTPILSDHET-----HQTYDVARRLGESLQLINILRDVGEDFENERI

3acxA/1-277 GYCYGVAGTVGEVLTPILSDHE-----THQTYDVARRLGESLQLINILRDVGEDFENERI

4e9uA/1-281 GYCYGVAGTVGEVLTPILSDHET-----HQTYDVARRLGESLQLINILRDVGEDFENERI

2zcoA/1-281 GYCYGVAGTVGEVLTPILSDHET-----HQTYDVARRLGESLQLINILRDVGEDFENERI

2zcsA/1-281 GYCYGVAGTVGEVLTPILSDHET-----HQTYDVARRLGESLQLINILRDVGEDFENERI

3acxA/1-277 GYCYGVAGTVGEVLTPILSDHE-----THQTYDVARRLGESLQLINILRDVGEDFENERI

4e9uA/1-281 GYCYGVAGTVGEVLTPILSDHET-----HQTYDVARRLGESLQLINILRDVGEDFENERI

2zcsA/1-270 GYCYGVAGTVGEVLTPILSDHET-----HQTYDVARRLGESLQLINILRDVGEDFENERI

4hd1A/1-258 DYCRYSADPVGRLVLGIFGCLD------DERARLSDATCTALQVANH--DIDRDLALGRI

3vj8A/1-290 KYCHYVAGLVGIGLSRLFSASEFEDPLVGEDTERANSMGLFLQKTNIIRDYLEDQQGGRE

2nd_struc_pred/1-351 HHHHHHHHHHHHHHHHHHCCCCCCCHCHHHHHHHHHHHHHHHHHHHHHHHHHHHHHCCCS

PSY_F/1-351 YLPQDELAQAGLSDEDIFAGKVTDKWRNFMKMQLKRARMFFDEAEKGVTELDAASRWPVW

2zcsA/1-281 YFSKQRLKQYEVDIAEVYQNGVNNHYIDLWEYYAAIAEKDFRDVMDQIKVFSIEAQPIIE

3acxA/1-277 YFSKQRLKQYEVDIAEVYQNGVNNHYIDLWEYYAAIAEKDFRDVMDQIKVFSIEAQPIIE

4e9uA/1-281 YFSKQRLKQYEVDIAEVYQNGVNNHYIDLWEYYAAIAEKDFRDVMDQIKVFSIEAQPIIE

2zcoA/1-281 YFSKQRLKQYEVDIAEVYQNGVNNHYIDLWEYYAAIAEKDFRDVMDQIKVFSIEAQPIIE

2zcsA/1-281 YFSKQRLKQYEVDIAEVYQNGVNNHYIDLWEYYAAIAEKDFRDVMDQIKVFSIEAQPIIE

3acxA/1-277 YFSKQRLKQYEVDIAEVYQNGVNNHYIDLWEYYAAIAEKDFRDVMDQIKVFSIEAQPIIE

4e9uA/1-281 YFSKQRLKQYEVDIAEVYQNGVNNHYIDLWEYYAAIAEKDFRDVMDQIKVFSIEAQPIIE

2zcsA/1-270 YFSKQRLKQYEVDIAEVYQNGVNNHYIDLWEYYAAIAEKDFRDVMDQIKVFSIEAQPIIE

4hd1A/1-258 YVPRADLEQFGATLDDIRARRATDGVRRCIALEVDRAQALFDEGRRLESLVPPRLARQLK

3vj8A/1-290 FWPQEVWSRYVKKLGDFAKPENIDLAVQCLNELITNALHHIPDVITYLSRLRNQSVFNFC

2nd_struc_pred/1-351 SCCHHHHHHCCCCHHHHHCCCCCHHHHHHHHHHHHHHHHHHHHHHHHHHHCCHHHHHHHH

PSY_F/1-351 ASLLLYRRILDEIEANDCNNFTKRAYVGKAKKIAALPLAYAKSILKASSSR

2zcsA/1-281 LAARIYIEILDEVRQANY-TLHERVFVEKRKKAKLFHEINSKY--------

3acxA/1-277 LAARIYIEILDEVRQANYT-LHERVFVEKRKKAKLFHEINSKY--------

4e9uA/1-281 LAARIYIEILDEVRQANYT-LHERVFVEKRKKAKLFHEINSKY--------

2zcoA/1-281 LAARIYIEILDEVRQANYT-LHERVFVEKRKKAKLFHEIN--------SKY

2zcsA/1-281 LAARIYIEILDEVRQANYT-LHERVFVEKRKKAKLFHEINSKY--------

3acxA/1-277 LAARIYIEILDEVRQANYT-LHERVFVEKRKKAKLFHEINSKY--------

4e9uA/1-281 LAARIYIEILDEVRQANY-TLHERVFVEKRKKAKLFHEINSKY--------

2zcsA/1-270 LAARIYIEILDEVRQANY-TLHERVFVEKRKKAKLFHEINSK---------

4hd1A/1-258 LYRLGGEAILAAIRRQGYNPF------------------------------

3vj8A/1-290 IPQVMAIATLAACY-NNQQVFKGAVKIRKGQAVTLMMDATNMPAVHRIPD-

2nd_struc_pred/1-351 HHHHHHHHHHHHHHHCCCCCCCCCCCCCHHHHHHHHHHHHHHHHHHHHCCC

**Figure S4. Alignment of *B. napus* PSY proteins with squalene synthase and carotenoid dehydrosqualene synthase templates.** 2zcs: *Staphylococcus aureus* Dehydrosqualene synthase complexed with BPH-700*;* 3acx: *Staphylococcus aureus* Dehydrosqualene synthase complexed with BPH-673*;* 4e9u: *Staphylococcus aureus* Dehydrosqualene synthase complexed with thiocyanate inhibitor*;* 2zco: *Staphylococcus aureus* Dehydrosqualene synthase*;* 4hdl: PpcA F15L mutant from *Geobacter sulfurreducens**;* 3vj8: *Homo sapiens* Squalene synthase*.*

**Figure S5. Tridimensional enzyme structure prediction of *B. napus* PSY homoelogous pairs.** Alpha-helices are shown in green and the putative active site (DXXXD) in purple with the four conserved aspartate residues shown as licorice representations [49].

**Table S1.** **Oligonucleotide primers used in this study**

| **Name** | **5'-sequence-3'** |
| --- | --- |
| ***BnaX.PSY* CDS cloning** | |
| BnaA.PSY.d-F1 | AGAGGAGTTCTTCTTCTCCTTCTTC |
| BnaA.PSY.d-R1 | CGGTTGATAGTCTTGCTTCTCTCAT |
| BnaC.PSY.f-F1 | CCCAATGAACAATCCTGGGTTAA |
| BnaA.PSY.c-F1 | CTTATGCTATTTCACTCTTCTGCC |
| BnaC.PSY.e-F1 | TTAATCCATTTGCAGGAAGGCC |
| BnaC.PSY.e-R1 | CAACCCTAAGCTTTGCCTTTAATC |
| Bna.PSY.15F | GCTCGAGTATGTCTTCTGTAGCAGT |
| Bna.PSY.8R | TTCGACCCTAAGCTTTTCCT |
| **5' RACE** | |
| BnaA.PSY.NR.b | CAGAAGAAGAGGAAGAAGTCCATGTTT |
| BnaA.PSY.GR.b | GCTACTACGCTCGAAGACACCACACTC |
| BnaA.PSY.NR.c | GTTCATTGGGTCTGGATTTGGAGAAGGA |
| BnaA.PSY.GR.c | CTGCGGCGTCTCATAACAGAAGAACTC |
| BnaA.PSY.NR.d | CTAGAACCCTTACCAACCCAGAATTGG |
| BnaC.PSY.NR.e | TGCAGGATGTGCTACTAAGCTTGAAGAG |
| BnaC.PSY.GR.e | TCCTGTGGTTTCTTCACATCGTCAAGAA |
| BnaC.PSY.NR.f | GGCTTCTTCAGATCATCAAGGTCCCTAA |
| BnaC.PSY.GR.f | CCCAACAAACTCAAACTCCCAGTAGTAG |
| BnaC.PSY.GR.G | GGGTTATCTCTCCTGCAGCAGGACTT |
| **3' RACE** | |
| BnaA.PSY.GF.b | CAAGCTGGTCTCTCAGATGAAGACATC |
| BnaA.PSY.NF.b | GCTAGCAGATGGCCGGTGTGGGCATCT |
| BnaA.PSY.GF.c | GCTCAAGCTGGTCTCTCAGATGAAGAT |
| BnaA.PSY.NF.c | GGCCAGTATGGGCATCGCTCCTATTA |
| BnaC.PSY.NF.f | CGATGTTCAGCCATTTAGAGACATGGTA |
| BnaC.PSY.GF.G | CCGTGGCCGTCCTTTCGATATGCTTGA |
| BnaC.PSY.NF.G | GACGCTGCTCTAGCTGATACAGTTGCT |
| ***BnaX.PSY* CDS without signal peptide for pETblue-1 cloning** | |
| BnaC.PSY.ayb-CHF | ATGGCAAGTCATGCAGGAGAGATAGC |
| BnaC.PSY.ayb-CHR | TTAAGTTGTTCCTCTTGAACTTGG |
| BnaA.PSY.c-CHF | ATGTCAAGCTTAGTCGTAAATCCTG |
| BnaA.PSY.c-CHR | CTCTTAACTTGAAACCTTTAGTACTG |
| BnaA.PSY.d-CHF | ATGTCAAGCTTAGTAGCAAGTCCTGCT |
| BnaA.PSY.d-CHR | TCATCTTGAACTTGAAGCCTTTAG |
| BnaC.PSY.e-CHF | ATGTCAAGCTTAGTAGCACATCCTGC |
| BnaC.PSY.e-CHR | CTCTTAACTTGAAACCTTTACTACTG |
| BnaC.PSY.f-CHF | ATGTCAAGTTTAGTAGCAAGTCCTGCT |
| BnaC.PSY.f-CHR | TCATCTTGAACTTGAAGCCTTTAG |
| **RT-PCR** | |
| PSYaSpecF | CAAAATTCTCTTCCAAGAGGAGGAGAAAAG |
| PSYaRev | AGCTGTTTGTTAACCAAAGCCGCTTGT |
| PSYbSpecF | AACAAAACAAAAAGTTGAGATTTTTCCATT |
| BnaPSY.NRb | CAGAAGAAGAGGAAGAAGTCCATGTT |
| PSYcSpecF3 | TTGGAGTTCTTCTGTTATGAGACGCCG |
| PSYcRev | CGCCATGGGAGTTATATGTGATGC |
| PSYdSpecF | TCTCTAGTGAGCTGCAGAAGGGGAAGA |
| PSYdRev | CTAACGCCATGGGAGTTATGTGTG |
| PSYeSpecF2 | GAGTTCTTCTTCTTCTGTAATGAGCTTT |
| PSYeRev2 | CCATGGGAGTTATATGTGATGCATTC |
| PSYfSpecF | CTTCTGTAATGAGCTGCAGAAGAAGATTAA |
| PSYfRev | CTTCCCATCTATCTAACGCCATGG |
| Bna.18SF | CAGACTGTGAAACTGCGAATGG |
| Bna.18SR | GCTAATGTATCCAGAGCGTAGGCT |
| Bna.ActinF | GAACTGGAATGGTGAAGGCTGGGTT |
| Bna.ActinR | TCTCTCAGCTCCGATGGTGATGACT |

**Table S2. BnaX.PSY protein sequence identity (%).**

| **Full length BnaX.PSY proteins (with signal peptide)** | | | | | | |
| --- | --- | --- | --- | --- | --- | --- |
| **SeqA** | **Name** | **Length** | **SeqB** | **Name** | **Length** | **%** |
| 1 | BnaC.PSY.a | 424 | 2 | BnaA.PSY.b | 423 | **98,8** |
| 1 | BnaC.PSY.a | 424 | 3 | BnaA.PSY.c | 414 | **89,4** |
| 1 | BnaC.PSY.a | 424 | 4 | BnaA.PSY.d | 418 | **87,4** |
| 1 | BnaC.PSY.a | 424 | 5 | BnaC.PSY.e | 421 | **90,4** |
| 1 | BnaC.PSY.a | 424 | 6 | BnaC.PSY.f | 416 | **87,3** |
| 1 | BnaC.PSY.a | 424 | 7 | AtPSY | 422 | **89,2** |
| 2 | BnaA.PSY.b | 423 | 3 | BnaA.PSY.c | 414 | **90,3** |
| 2 | BnaA.PSY.b | 423 | 4 | BnaA.PSY.d | 418 | **88,3** |
| 2 | BnaA.PSY.b | 423 | 5 | BnaC.PSY.e | 421 | **91,3** |
| 2 | BnaA.PSY.b | 423 | 6 | BnaC.PSY.f | 416 | **88,4** |
| 2 | BnaA.PSY.b | 423 | 7 | AtPSY | 422 | **89,9** |
| 3 | BnaA.PSY.c | 414 | 4 | BnaA.PSY.d | 418 | **86,7** |
| 3 | BnaA.PSY.c | 414 | 5 | BnaC.PSY.e | 421 | **93,6** |
| 3 | BnaA.PSY.c | 414 | 6 | BnaC.PSY.f | 416 | **85,6** |
| 3 | BnaA.PSY.c | 414 | 7 | AtPSY | 422 | **87,2** |
| 4 | BnaA.PSY.d | 418 | 5 | BnaC.PSY.e | 421 | **86,9** |
| 4 | BnaA.PSY.d | 418 | 6 | BnaC.PSY.f | 416 | **95,9** |
| 4 | BnaA.PSY.d | 418 | 7 | AtPSY | 422 | **88,5** |
| 5 | BnaC.PSY.e | 421 | 6 | BnaC.PSY.f | 416 | **86,4** |
| 5 | BnaC.PSY.e | 421 | 7 | AtPSY | 422 | **87,1** |
| 6 | BnaC.PSY.f | 416 | 7 | AtPSY | 422 | **87,6** |
| **Mature BnaX.PSY proteins (without signal peptide)** | | | | | | |
| **SeqA** | **Name** | **Length** | **SeqB** | **Name** | **Length** | **%** |
| 1 | BnaC.PSY.a | 349 | 2 | BnaA.PSY.b | 349 | **99,4** |
| 1 | BnaC.PSY.a | 349 | 3 | BnaA.PSY.c | 348 | **91,8** |
| 1 | BnaC.PSY.a | 349 | 4 | BnaA.PSY.d | 351 | **92,2** |
| 1 | BnaC.PSY.a | 349 | 5 | BnaC.PSY.e | 348 | **91,5** |
| 1 | BnaC.PSY.a | 349 | 6 | BnaC.PSY.f | 351 | **91,6** |
| 1 | BnaC.PSY.a | 349 | 7 | AtPSY | 366 | **90,2** |
| 2 | BnaA.PSY.b | 349 | 3 | BnaA.PSY.c | 348 | **92,4** |
| 2 | BnaA.PSY.b | 349 | 4 | BnaA.PSY.d | 351 | **92,7** |
| 2 | BnaA.PSY.b | 349 | 5 | BnaC.PSY.e | 348 | **92,1** |
| 2 | BnaA.PSY.b | 349 | 6 | BnaC.PSY.f | 351 | **92,2** |
| 2 | BnaA.PSY.b | 349 | 7 | AtPSY | 366 | **90,7** |
| 3 | BnaA.PSY.c | 348 | 4 | BnaA.PSY.d | 351 | **91,5** |
| 3 | BnaA.PSY.c | 348 | 5 | BnaC.PSY.e | 348 | **96** |
| 3 | BnaA.PSY.c | 348 | 6 | BnaC.PSY.f | 351 | **91** |
| 3 | BnaA.PSY.c | 348 | 7 | AtPSY | 366 | **88,8** |
| 4 | BnaA.PSY.d | 351 | 5 | BnaC.PSY.e | 348 | **91** |
| 4 | BnaA.PSY.d | 351 | 6 | BnaC.PSY.f | 351 | **98,9** |
| 4 | BnaA.PSY.d | 351 | 7 | AtPSY | 366 | **89,5** |
| 5 | BnaC.PSY.e | 348 | 6 | BnaC.PSY.f | 351 | **90,4** |
| 5 | BnaC.PSY.e | 348 | 7 | AtPSY | 366 | **88,3** |
| 6 | BnaC.PSY.f | 351 | 7 | AtPSY | 366 | **88,9** |

**Table S3. Ka/Ks.**

| **Gene 1** | **Gene 2** | **Ks** | **Ka** | **Ka/Ks** |
| --- | --- | --- | --- | --- |
| ***BnaC.PSY.a*** | ***BnaA.PSY.b*** | 0,1279 | 0,0045 | 0,03518374* |
| ***BnaC.PSY.a*** | ***BnaA.PSY.c*** | 0,275 | 0,0381 | 0,13854545 |
| ***BnaC.PSY.a*** | ***BnaA.PSY.d*** | 0,2304 | 0,0341 | 0,14800347 |
| ***BnaC.PSY.a*** | ***BnaC.PSY.e*** | 0,2675 | 0,0375 | 0,14018692 |
| ***BnaC.PSY.a*** | ***BnaC.PSY.f*** | 0,2505 | 0,0376 | 0,1500998 |
| ***BnaC.PSY.a*** | ***AtPSY*** | 0,3667 | 0,0393 | 0,10717208** |
| ***BnaA.PSY.b*** | ***BnaA.PSY.c*** | 0,2652 | 0,0334 | 0,12594268 |
| ***BnaA.PSY.b*** | ***BnaA.PSY.d*** | 0,2479 | 0,03 | 0,12101654 |
| ***BnaA.PSY.b*** | ***BnaC.PSY.e*** | 0,2783 | 0,0317 | 0,11390586 |
| ***BnaA.PSY.b*** | ***BnaC.PSY.f*** | 0,2736 | 0,0323 | 0,11805556 |
| ***BnaA.PSY.b*** | ***AtPSY*** | 0,3674 | 0,0335 | 0,09118127** |
| ***BnaA.PSY.c*** | ***BnaA.PSY.d*** | 0,2787 | 0,044 | 0,15787585 |
| ***BnaA.PSY.c*** | ***BnaC.PSY.e*** | 0,1634 | 0,0236 | 0,14443084* |
| ***BnaA.PSY.c*** | ***BnaC.PSY.f*** | 0,2439 | 0,0487 | 0,199672 |
| ***BnaA.PSY.c*** | ***AtPSY*** | 0,374 | 0,051 | 0,13636364** |
| ***BnaA.PSY.d*** | ***BnaC.PSY.e*** | 0,2712 | 0,0422 | 0,15560472 |
| ***BnaA.PSY.d*** | ***BnaC.PSY.f*** | 0,1184 | 0,0157 | 0,13260135* |
| ***BnaA.PSY.d*** | ***AtPSY*** | 0,3646 | 0,03 | 0,08228195** |
| ***BnaC.PSY.e*** | ***BnaC.PSY.f*** | 0,2417 | 0,0469 | 0,18907737 |
| ***BnaC.PSY.e*** | ***AtPSY*** | 0,3713 | 0,0457 | 0,09641799** |
| ***BnaC.PSY.f*** | ***AtPSY*** | 0,4131 | 0,0358 | 0,08666183** |

*homoelogues;**orthologues
